# Nutritional Status of Patients with Facioscapulohumeral Muscular Dystrophy

**DOI:** 10.3390/nu15071673

**Published:** 2023-03-30

**Authors:** Sedda Amzali, Vinicius Dias Wilson, Sébastien Bommart, Marie-Christine Picot, Simon Galas, Jacques Mercier, Patrick Poucheret, Jean-Paul Cristol, Sandrine Arbogast, Dalila Laoudj-Chenivesse

**Affiliations:** 1 PhyMedExp, Université de Montpellier, INSERM, CNRS, CHU de Montpellier, 34295 Montpellier, Francejacques.mercier@umontpellier.fr (J.M.); jp-cristol@chu-montpellier.fr (J.-P.C.); sandrine.arbogast@inserm.fr (S.A.); 2 Departamento de Educação Física, Centro Universitário Estácio de Belo Horizonte, Belo Horizonte 30411-052, Minas Gerais, Brazil; 3 Pró-Reitoria de Assuntos Comunitários e Estudantis, Universidade Federal dos Vales do Jequitinhonha e Mucuri, Diamantina 39100-000, Minas Gerais, Brazil; 4 Service de Radiologie, Hôpital Arnaud-de-Villeneuve, CHU de Montpellier, 34295 Montpellier, France; 5 Clinical Research and Epidemiology Unit (Department of Medicale Information), Centre d’Investigation Clinique 1411 INSERM, CHU Montpellier, Univ Montpellier, CEDEX 5, 34295 Montpellier, France; 6 Institut des Biomolecules Max Mousseron (IBMM), Centre National de Recherche Scientifique (CNRS), University of Montpellier, ENSCM, 34000 Montpellier, France; simon.galas@umontpellier.fr; 7 Department of Clinical Physiology, CHU of Montpellier, 34295 Montpellier, France; 8 Qualisud, Université de Montpellier, CIRAD, Institut Agro, IRD, Avignon Université, Université de La Réunion, 34000 Montpellier, France; patrick.poucheret@umontpellier.fr; 9 Department of Biochemistry, University Hospital of Montpellier, 34295 Montpellier, France

**Keywords:** facioscapulohumeral muscular dystrophy, metabolism, nutrition, oxidative stress

## Abstract

In patients with facioscapulohumeral muscular dystrophy (FSHD), a rare genetic neuromuscular disease, reduced physical performance is associated with lower blood levels of vitamin C, zinc, selenium, and increased oxidative stress markers. Supplementation of vitamin C, vitamin E, zinc, and selenium improves the quadriceps’ physical performance. Here, we compared the nutritional status of 74 women and 85 men with FSHD. Calorie intake was lower in women with FSHD than in men. Moreover, we assessed vitamin C, vitamin E, zinc, copper, and selenium intakes in diet and their concentrations in the plasma. Vitamin E, copper, and zinc intake were lower in women with FSHD than in men, whereas plasma vitamin C, copper levels, and copper/zinc ratio were higher in women with FSHD than in men. The dietary intake and plasma concentrations of the studied vitamins and minerals were not correlated in both sexes. A well-balanced and varied diet might not be enough in patients with FSHD to correct the observed vitamin/mineral deficiencies. A low energy intake is a risk factor for suboptimal intake of proteins, vitamins, and minerals that are important for protein synthesis and other metabolic pathways and that might contribute to progressive muscle mass loss. Antioxidant supplementation and higher protein intake seem necessary to confer protection against oxidative stress and skeletal muscle mass loss.

## 1. Introduction

Facioscapulohumeral muscular dystrophy (FSHD) is a disabling inherited muscle disease that affects children and adults. FSHD is one of the most common types of muscular dystrophy, with a prevalence ranging from 3.2 to 4.6 per 100,000 in Europe [1,2,3]. This autosomal dominant disorder is characterized by progressive and asymmetric weakness of facial and scapular muscles [4]. Lower extremity involvement, which may be more common than previously appreciated [5], eventually leads to wheelchair use [4]. The muscle weakness pattern is often asymmetrical, and its progression rate and extent may vary considerably, with sudden periods of unexplained rapid disease progression [6,7]. The major form of FSHD is genetically linked to deletions in chromosome 4q35 within the *D4Z4* repeat array [8], associated with DNA hypomethylation [9,10,11] that leads to reduced heterochromatinization of the region. This results in the transcription of usually repressed genes, such as the double-homeodomain transcription factor *DUX4* believed to cause the disease through a toxic gain-of-function mechanism [12,13]. In myoblasts, aberrant DUX4 expression initiates a large signaling cascade that leads to muscle cell differentiation defects, oxidative stress, and muscle atrophy [12,14,15,16], which are key features of FSHD [17]. A recent study demonstrated that oxidative stress increases DUX4 expression in FSHD myocytes through a DNA damage response signaling pathway [18]. In addition to *DUX4* expression, other genes in the 4q35 region (e.g., *DUX4c*, *FRG2*, and *FRG1*) could be activated and act as modifiers [19]. Despite major progress in the understanding of the genetic basis of FSHD, the specific pathogenic mechanisms remain unclear, and no curative treatment is available.

Growing evidence suggests that oxidative stress may contribute to FSHD pathology [20,21,22,23,24,25,26]. This hypothesis is supported by the observation that in FSHD muscle biopsies, enzymes involved in oxidative stress are deregulated, and oxidative damage is increased [22]. Ex vivo experiments showed that primary myoblasts from patients with FSHD are more sensitive to exogenous pro-oxidants [25,26]. Moreover, several genes involved in oxidative stress susceptibility are affected by DUX4 derepression. The acute cell toxicity mediated by DUX4 overexpression in mouse C2C12 myoblasts is alleviated by addition of antioxidants, such as ascorbic acid and vitamin E, to the culture medium [27] However, in FSHD myocytes, the activation by oxidative stress of a DNA damage response signaling pathway induces an increase in DUX4 expression [18]. Collectively, these findings suggest that in FSHD, oxidative damage might lead to muscle injury. The exact processes underlying the increase in oxidative stress in FSHD remain unelucidated. In a previous study, we reported that reduced physical performance in patients with FSHD is associated with an important redox unbalance and oxidative stress in the blood [22]. Specifically, analysis of systemic oxidative stress markers showed that in most patients with FSHD, oxidative damage is increased (higher lipid peroxide and oxidized DNA levels), and the concentration of vitamin C and essential elements, particularly selenium (a cofactor for glutathione peroxidase involved in the elimination of lipid peroxides) and zinc (a copper/zinc dismutase cofactor involved in superoxide elimination), is decreased compared with healthy controls [22]. In addition, the ratio between reduced and oxidized glutathione (GSSG) is strongly decreased in most patients as a consequence of GSSG accumulation. Glutathione (GSH) can interact directly with activated oxygen species, but its main antioxidant function is as a substrate of glutathione peroxidase, the enzyme that eliminates lipid peroxides [28,29]. In oxidative stress, GSH is usually depleted, but not in FSHD patients, suggesting a decreased efficiency of the enzymes that use it. Moreover, low vitamin C to ascorbyl radical ratios in patients with FSHD indicate that GSH is not properly used to regenerate vitamin C [30]. Therefore, we hypothesized that, in FSHD, insufficient intake of antioxidant vitamins and minerals may reduce the body’s capacity to regulate free radical insults, leading to oxidative stress that could affect skeletal muscle contractibility and function. In a previous randomized placebo-controlled trial (*n* = 53 patients with FSHD), we found that antioxidant supplementation (500 mg vitamin C, 400 mg vitamin E, 25 mg zinc, and 200 µg selenium) improved maximal voluntary contraction of both quadriceps by enhancing the antioxidant defenses, and by reducing oxidative stress [23]. However, in this previous study, we did not investigate the dietary intake of these micronutrients. Nutrition is one of the most important modifiable lifestyle factors implicated in chronic disease development [31,32]. According to a World Health Organization (WHO) estimate, 80% of chronic diseases are due to lifestyle- and diet-related factors [33]. Therefore, improving lifestyle and diet by adopting a balanced and healthy diet is essential for the prevention of many diseases [34]. Nutritional knowledge is a major factor in the selection of a healthy and nutritious diet [35]. The importance of an adequate nutritional status has become clear in recent decades, with direct links to improved muscle function [36].

Therefore, the first aim of our present study was to assess the nutritional intakes in 159 patients (74 women and 85 men) with FSHD and to identify differences between sexes and relative to the recommended daily recommended nutrient intakes (RNI), published by the French National Agency for Food, Environmental and Occupational Health Safety (ANSES) in 2016. The second aim was to assess vitamin C, vitamin E, zinc, copper, and selenium dietary intakes and their plasma concentrations.

## 2. Materials and Methods

### 2.1. Study Design

This study included patients from a randomized double-blind placebo-controlled study (NCT01596803) recruited between May 2010 and April 2012 and patients with FSHD routinely followed at the Clinical Physiology Department, Montpellier University Hospital (France), between April 2015 and June 2017 (NCT02622438). Inclusion criteria were as follows: between one and nine D4Z4 repeat units and a family history of FSHD; and age between 14 and 75 years. Evaluations were performed as follows: anthropometric assessment, physical activity level (PAL) and nutritional status (macronutrients, micronutrients, and energy intake), antioxidant status and oxidative stress markers, and quadriceps maximal voluntary contraction (MVC_Q_).

Furthermore, magnetic resonance imaging (MRI) was performed in the framework of the ancillary study to the randomized double-blind placebo-controlled study (NCT01596803) entitled “Effects Antioxidants Supplementation on Muscular Function Patients Facioscapulohumeral Dystrophy (FSHD)” [23]. After protocol amendment approval, 32 patients with FSHD from the double-blind, placebo-controlled, randomized trial (NCT01596803) and 7 sedentary (i.e., <1 h of physical activity per week) healthy controls were also included for additional analyses. For this ancillary study, thigh muscle and fat volumes were assessed by magnetic resonance imaging (MRI) in patients who signed an additional consent form. Inclusion and exclusion criteria for the ancillary study were the same as for the main trial [23]: age between 18 and 60 years; D4Z4 repeat units between four and nine with a positive family history for FSHD; and no HIV and/or hepatitis. Exclusion criteria included confinement to a wheel chair, smoking, concomitant comorbidity (e.g., cardiorespiratory diseases, diabetes), or being on medication (including mineral or vitamin supplements and/or other antioxidants). Some new exclusion criteria for the MRI evaluation were added as follows: patients with claustrophobia, cardiac pacemaker or defibrillator, cochlear, otologic or ear implants, and foreign intraocular metal bodies.

### 2.2. Clinical and Anthropometric Data

Anthropometric measurements were performed by trained hospital staff in a standardized way according to the WHO guidelines. Body weight (kg) was measured using a digital scale to the nearest 0.1 kg, and height (m) was measured using a standardized stadiometer to the nearest 0.1 cm. Body mass index (BMI) was calculated as body weight (kg)/height^2^ (m^2^) [37]. Patients were categorized as underweight (BMI < 18.5 kg/m^2^), normal weight (BMI [18.5–24.9 kg/m^2^]), overweight (BMI [25–29.9 kg/m^2^]), or obese (BMI ≥ 30 kg/m^2^) according to the WHO [38]. Waist and hip circumferences were measured using a standardized procedure. The reference waist circumference values were 83 to 98 cm for men and 78 to 91 cm for women. The minimum normal waist circumference cut-off values were 94 cm for men and 80 cm for women. The reference hip circumference values were 94 to 105 cm for men and 97 to 108 cm for women. The waist/hip ratio (WHR) (0.87 to 0.99 for men, 0.76 to 0.84 for women) was evaluated in relation to the clinical risk thresholds of WHR  > 1 for men and WHR  > 0.9 for women [39].

### 2.3. Nutritional Analysis

The patients’ food intake was assessed using a self-reported three-day dietary record completed on three non-consecutive days (two weekdays and one weekend day), as performed in several dietary surveys [40]. Participants were instructed to record and provide detailed descriptions of their food and drink consumption (food type, ingredients, number of meals, and cooking method). Dietary records were reviewed with a nutritionist to clarify the amounts of food ingested.

To estimate the intake of energy, macronutrients and micronutrients, all food data collected were entered and analyzed using the computerized food survey management software GENI©, developed by MICRO 6© (Version 9.6; GENI, Villers-Lès-Nancy, France). The software uses the French reference table on the nutritional composition of foods, established by the Food Quality Information Centre (CIQUAL 2016), which is one of the most complete tables in Europe with more than 2600 generic foods consumed in France and 61 constituents.

This software allowed the quantification of the total energy intake, energy from macronutrients (percentage of energy from proteins, fat, and carbohydrates), and nutrient dietary intake (vitamins C and E, copper, zinc, selenium, and cholesterol). The daily intakes of vitamin C and E, copper, zinc, and selenium were compared with the daily RNI, published by ANSES in 2016 [41], to assess their adequacy. For cholesterol, the WHO recommendations [42] were used. Nutrient intakes were classified as adequate, inadequate, or excessive by comparing the mean intake to the RNI values.

For the energy analysis of macronutrients and micronutrients, the mean daily intakes were calculated for each patient by averaging the three-day survey data.

### 2.4. Antioxidant Status and Oxidative Stress Markers

The antioxidant status and stress markers were evaluated in 32 patients and 7 healthy controls as previously described [23]. Venous blood was collected from each patient after overnight fasting to assess the plasma concentration of vitamins C and E (as alpha-tocopherol), copper, zinc, selenium, reduced (GSH) and oxidized glutathione (GSSG), glutathione peroxidase (GSH-Px), superoxide dismutase (CuZn-SOD), and a marker of lipid peroxidation (lipid peroxides). The detection of urinary 8-hydroxyguanosine, 8-OH-dG, a marker of DNA oxidation (oxidized DNA) levels, was normalized to creatinine levels in urine. Each parameter was routinely determined at Liege University Hospital, Belgium, as previously described [22]. The reference intervals of the mean values of antioxidant and oxidative stress markers were obtained from a large healthy population [43,44].

### 2.5. Muscle Volume by MRI

Patients (*n* = 32) and healthy controls (*n* = 7) underwent MRI examinations as previously described [27], using the same 1.5 Tesla MRI apparatus (Magnetom Area Avento, Siemens Medical, Erlangen, Germany) and “lower limbs” surface antennas without contrast agent. Subjects were in supine position, lower limbs at rest. The acquisition of T1-weighted three-dimensional (3D) flash sequences allowed three orthogonal plane reconstructions and tissue characterization. Thigh muscles were visually identified bilaterally. The region of interest included quadriceps muscles (rectus femoris, vastus lateralis, vastus intermedius, and vastus medialis) and posterior muscles, including the medial muscles (pectineus, gracilis, and the three adductors: long, short, and wide), hamstrings (semi-membranous, semi-tendon, and biceps femoris), and sartorius. Quadricep and posterior muscles were manually outlined on both thighs in all selected slices by the same investigator, from the little trochanter to the proximal extremity of the patella. Semi-automated morphological analyses were performed using the Myrian software (Intrasense, Montpellier, France). Post-processing was carried out using the thresholding technique and contour recognition to quantify the volumes of morphologically healthy muscle and fatty degeneration. Results were expressed as follows: muscle volume of quadriceps and posterior muscles (MV), fat volume (FV_Q_), total volume (TV_Q_), muscle volume percentage (%MV_Q_), and muscle fat percentage (%FV_Q_). The lipid ratio (RL) was determined with the formula:

RL = (ö[(Muscle Signal)^2^ − (Noise Signal)^2^]/ö[(Bone Marrow Signal)^2^ − (Noise Signal)^2^]) × 100.

### 2.6. Daily Physical Activity Level

Each patient’s daily PAL was evaluated using the Voorrips’ physical activity self-report questionnaire [45]. The questionnaire scores the past year’s household activities, sports activities, and other leisure-time physical activities and gives an overall physical activity score. Patients were asked to describe the activity type, number of hours per week, and period of the year in which the activity was normally performed. All activities were classified according to posture and movement. This questionnaire is a reliable and valid method to classify the PAL of older participants as high (score > 16.4), moderate (score between 9.4 and 16.4), or low (score < 9.4). The activity factors corresponding to low, moderate, and high PAL were 1.57, 1.78, and 2.10 in men and 1.56, 1.64, and 1.82 in women, respectively [46].

### 2.7. Quadriceps Maximal Voluntary Contraction

Quadriceps maximal voluntary contraction (MVC_Q_ (kg)) was assessed on an adapted exercise bench (Kettler, Germany) connected to a computer interface (Biopac, Acknowledge, France), as previously described [22,24,25]. Patients were instructed to remain seated (hips and knees at 90°) and to perform a contraction at their highest force for about 6s. To ensure the maximum effort, verbal encouragement was given. For each limb, three to five repetitions were performed (less than 10% variability) to calculate the mean MVC_Q_ and to determine the lower limb laterality. The thigh with the highest MVC_Q_ was considered the dominant thigh (MVC_QD_) and the other the non-dominant thigh (MVC_QND_).

### 2.8. Statistical Analyses

For all parameters (demographic, clinical, anthropometric, energy, nutrient intakes and their blood levels, PAL, and muscle strength), data were described as mean and standard deviation (SD) or median and interquartile range (IQR). For categorical variables, numbers (*n*) and percentages (%) were used. Data were tested for normality using the Shapiro–Wilk test. Depending on the normality of the parameters, differences between men and women were investigated with the Student’s *t*-test or the non-parametric Mann–Whitney Rank Sum test. The relationships between dietary intakes and the other studied parameters were determined using Spearman correlations. *p* values ≤ 0.05 were considered significant. To verify the effect size in the comparisons between men and women in the group with 32 patients, Cohen’s d was calculated, and the values of d were considered small if 0.20 ≤ d < 0.50; 0.50 ≤ d < 0.80 and large if d ≥ 0.80 [47]. All statistical analyses were performed with the SigmaPlot 14 software.

## 3. Results

Among the 132 patients with FSHD routinely followed at the Clinical Physiology Department, Montpellier University Hospital (France), between April 2015 and June 2017 (NCT02622438), only 106 met the inclusion criteria and had genetically confirmed FSHD type 1. The other 26 patients were excluded for the following reasons: consent withdrawal (*n* = 1), not FSHD type 1 (normal 4q35 allele) (*n* = 6), missing three-day dietary record (*n* = 14), supplemented with antioxidants before inclusion (*n* = 5). Additionally, 53 patients were also included from both arms of a double-blind and placebo-controlled, randomized trial (NCT01596803) (total *n* = 159 patients). For the MRI study, 32 of the 53 patients in the randomized trial and 7 sedentary (i.e., less than 1h of physical activity per week) healthy controls were evaluated.

### 3.1. Characteristics of Patients with FSHD

Table 1 shows the baseline anthropometric data of the 159 patients with FSHD (85 men and 74 women). In patients with FSHD, the mean number of D4Z4 repeat units (5.7 ± 1.8 in men and 5.6 ± 1.8 in women), the mean age (43.7 ± 14.7 years in men and 41.7 ± 14.5 years in women), and the mean BMI (23.8 ± 3.7 kg/m^2^ in men and 22.8 ± 4.8 kg/m^2^ in women) were comparable between sexes. Conversely, men were taller and heavier than women, and had a higher WHR due to their significantly larger waist circumference (90.2 ± 14.8 cm in men, 79.2 ± 13.4 cm in women; *p* < 0.001). When we categorized our population of patients with FSHD according to their age (young adults (14–30 years old), adults (31–50 years old), middle-aged (51–64 years old), and seniors (65–75 years old)), we confirmed the results obtained in all patients except for seniors (65–75 years old) (Table 2).

Supplementary Table S1 shows the baseline anthropometrics of the 32 patients with FSHD (20 men and 12 women) and 7 healthy controls (4 men and 3 women). Patients with FSHD and healthy controls did not differ in age, body weight, height, or BMI.

### 3.2. Dietary Energy Intake in 159 Patients with FSHD

The mean daily calorie intake (CI) was lower in all patients with FSHD (both sexes) than their respective recommended daily intake ranges (2470–2730 kcal/day for men and 1995–2205 kcal/day for women) (Table 3), and was lower in women than in men (1652.31 ± 342.6 kcal vs. 1914.4 ± 453.6 kcal, *p* < 0.001). Similarly, the mean CI was lower in women than in men with normal weight (1653.2 ± 400.7 vs. 1925.6 ± 464.9, *p* = 0.004) (Table 4). However, the mean CI was comparable among patients who were underweight, overweight, and obese (BMI) (Table 4). A more detailed analysis of each patient’s CI revealed that the daily CI was adequate in only 10.7% of men and 8.1% of women, inadequate in 84.5% of men and 83.8% of women, and above the recommendations in 4.8% of men and 8.1% of women (Table 3).

The mean percentage of energy from proteins, fat, and carbohydrates was comparable between sexes. However, the mean kilocalories from proteins, lipids and carbohydrates were significantly higher in men than in women (342.6 ± 89.2 vs. 304 ± 77.2, *p* = 0.004; 683.2 ± 191.9 vs. 626 ± 176.7, *p* = 0.05; 797.0 ± 257.3. vs. 674.2 ± 193.6, *p* = 0.001, respectively) (Table 3).

Furthermore, the protein intake (g/kg body weight/d) was comparable between men and women (Table 3). While the protein/energy ratio was adequate in 51.2% of men and 58.5% of women and excessive in 47.6% of men and 39.2% of women, protein intake (g/kg body weight/d) was adequate in 39.3% of men and 48.7% of women and lower than recommended in 58.3% of men and 45.9% of women (Table 3). The fat/energy ratio was adequate in 28.6% of men and 28.4% of women, higher in 34.5% of men and 41.9% of women, and lower than the recommended safe level in 36.9% of men and 29.7% of women. The carbohydrate/energy ratio was adequate in 59.5% of men and 60.8% of women and inadequate in 32.1% of men and 35.1% of women. While the protein/energy and carbohydrate/energy ratios were comparable in the four BMI categories (Table 4), the fat/energy ratio was significantly higher in women than in men with overweight (42.0 ± 5.8 vs. 37.6 ± 5.9, *p* = 0.029).

Calorie intake is influenced by many variables, including age. In this study, CI was higher in men than in women only among adults (*p* = 0.001) (Table 5). The daily CI was inadequate in young adult, adult, and middle-aged men and women (men: 94.4%, 89.5%, 87%; women: 70.6%, 84.2%, 84.6%, respectively) (Table 5). In senior men, daily CI was inadequate at 16.7% and excessive at 33.3%. In senior women, daily CI was inadequate at 60% and excessive at 33.3%. The mean percentage of energy from proteins, fat, and carbohydrates were similar among men and women of each age group. However, the mean kilocalories from proteins and lipids were only significantly higher in adult men than in women (*p* = 0.004; *p* = 0.041, respectively) (Table 5). Furthermore, the mean kilocalories from carbohydrates were significantly higher in adult and middle-aged men than in women (*p* = 0.008; *p* = 0.035, respectively). Furthermore, the protein intake (g/kg body weight/d) was similar amongst men and women of each age group. In men, the protein/energy ratio was adequate in 55.3% of middle-aged adults and 50% of seniors. In women, it was adequate in 47% of young adults, 58% of adults, 38.5% of middle-aged adults, and 66.7% of seniors. It was only excessive in young and middle-aged adult men (55.6% and 52.2%, respectively). Protein intake (g/kg body weight/d) was adequate in adult, middle age, and senior men and women (men: 84.2%, 87%, 66.7%; women: 86.8%, 69.2%, 66.7%, respectively) and lower than the recommended safe level respectively in 55.6% and 76.5% of young adult men and women (Table 5). The fat/energy ratio was adequate in 27.8% and 23.1% of young men and women, respectively. It was lower in 38.9% of men and 53.8% of women, and higher than the recommended safe level in 33.3% of men and 23.1% of women among young adults. In adults, the fat/energy ratio was adequate in 18.4% of men and 35.1% of women and inadequate in 36.8% of men and 29.7% of women. Furthermore, in middle-aged adults, the fat/energy ratio was adequate in 47.8% of men and 36.4% of women and inadequate in 34.8% of men and 27.3% of women. In seniors, the fat/energy ratio was adequate in 16.7% of men and 33.3% of women and inadequate in 33.3% of men and 33.3% of women. It was higher than the recommended safe level in 50% of men and 33.3% of women. The carbohydrate/energy ratio was adequate in 27.8% and 29.4% of men and women, respectively, and inadequate in 61.1% of men and 47.1% of women among young adults. In adults, the carbohydrate/energy ratio was adequate in 39.1% of men and 21.1% of women and inadequate in 34.8% of men and 38.4% of women. In middle-aged adults, the carbohydrate/energy ratio was adequate in 78.1% of men and 69.2% of women and inadequate in 21.7% of men and 30.8% of women. In seniors, the carbohydrate/energy ratio was respectively adequate and inadequate in 50% of men and women.

In the large population, the mean PAL was significantly higher in men than in women (1.65 ± 0.16 vs. 1.57 ± 0.02, *p* < 0.001) (Table 3). According to age, the mean PAL was significantly higher only in young and adult men than in women (Table 5).

When the 159 patients were categorized according to their PAL (low and moderate), CI was higher in men with low PAL than in women (1858.47 ± 415.7 vs. 1615.6 ± 350.5, *p* < 0.001) but no between-sex difference was observed in patients with moderate PAL (1924.1 ± 510.6 vs. 1923.4 ± 446.2, *p* = 0.998) (Table 6).

### 3.3. Micronutrient Dietary Intakes in 159 Patients with FSHD

In all patients with FSHD (*n* = 159), the dietary intakes of selenium, copper, the copper/zinc (Cu/Zn) ratio, cholesterol, and the vitamin C/vitamin E (VitC/VitE) ratio were adequate in 91.8%, 64.3%, 66.5%, 57.6%, and 53.8%, respectively. However, the dietary intakes of zinc, vitamin C, vitamin E, and the vitamin E/cholesterol (VitE/Chol) ratio were below the RNI recommendations in 84.8%, 70.9%, 69.6%, and 57%, respectively (Table 7).

Analysis of the micronutrient dietary intake in men and women with FSHD showed no difference in the dietary intakes of selenium, the Cu/Zn ratio, cholesterol, the VitE/Chol ratio, vitamin C, and the VitC/VitE ratio (Table 7). Conversely, the dietary intakes of vitamin E, copper, and zinc were significantly higher in men than in women (*p* = 0.029; *p* = 0.011; *p* = 0.005, respectively). The dietary intake of selenium was adequate in 92.9% of men and in 90.6% of women. The dietary intakes of vitamin C, vitamin E, and the VitC/VitE ratio were below the RNI recommendations in 70.2%, 69%, and 44% of men and 71.6%, 70.3%, and 48.6% of women, respectively. Similarly, the dietary intakes of zinc and the VitE/Chol ratio were below the RNI recommendation in 86.9% and 57.2% of men and in 82.4% and 56.8% of women. Moreover, the dietary intake of copper was below the RNI recommendation in 60.7% of men but adequate in 93.2% of women. Finally, the dietary cholesterol intake was above the RNI recommendations for 47.6% of men and 36.5% of women. According to age, analysis of the micronutrient dietary intake in patients with FSHD showed no difference between men and women at all ages in the dietary intakes of vitamin C, the VitC/VitE ratio, cholesterol, the VitE/Chol ratio, and the Cu/Zn ratio, as observed between men and women from the larger population (*n* = 159) (Table 8). However, in adults, the dietary intakes of vitamin E, zinc, and selenium were significantly higher in men than in women (*p* = 0.040; *p* = 0.016; *p* = 0.046, respectively) (Table 8). Additionally, the dietary intakes of copper were significantly higher in men than in women in both adults and middle-aged adults (*p* = 0.007; *p* = 0.034).

### 3.4. Micronutrient Plasma Concentrations in 159 Patients with FSHD

The mean plasma concentrations of vitamin E, cholesterol, copper, zinc, vitamin E levels normalized to lipid cholesterol levels [48], and the VitE/Chol ratio were within their reference intervals in patients with FSHD (Table 9). Conversely, the plasma concentrations of vitamin C, selenium, Cu/Zn, and VitC/VitE ratios were below the lower limit of their reference intervals in 53.2%, 62.2%, 39.1%, and 73.3% of patients with FSHD, respectively. They were above the upper limit of their reference intervals in 14.3%, 6.4%, 36.5%, and 18% of patients with FSHD, respectively.

A comparison of the plasma concentrations of the same micronutrients showed no difference between sexes in the VitE/Chol ratio, cholesterol, zinc, or selenium levels (Table 9). Conversely, the plasma levels of vitamin C and copper and the VitC/VitE and the Cu/Zn ratios were different between men and women (8.3 ± 3.2 vs. 9.99 ± 3.8, *p* = 0.003; 0.86 ± 0.2 vs. 1.21 ± 0.37, *p* < 0.001; 0.68 ± 0.4 vs. 0.86 ± 0.4, *p* = 0.003; and 0.94 ± 0.23 vs. 1.42 ± 0.57, *p* = 0.001, respectively). The mean plasma concentrations of vitamin C and vitamin E were below the lower limit of their reference intervals in 64.6% and 9.5% of men and in 40.3% and 5.6% of women. They were above the upper limit of their reference intervals in 7.3% and 10.7% of men and in 22.2% and 4.1% of women, respectively. The vitamin C/vitamin E and vitamin E/cholesterol ratios were below the lower limit of their reference intervals in 80% and 2.4% of men and in 65.7% and 0% of women, respectively. They were above the upper limit of their reference intervals in 11.2% and 36.6% of men and in 25.7% and 26.8% of women, respectively. The mean copper and zinc plasma levels were below the lower limit of their reference intervals in 16.9% and 7.1% of men and in 5.5% and 9.6% of women, respectively, and above the upper limit of their reference intervals 0% and 6% of men and in 16.4% and 5.5% of women, respectively. The mean copper/zinc ratio was below the lower limit of its reference intervals in 57.8% of men and 17.8% of women and above the upper limit of its reference intervals in 14.5% of men and 61.6% of women. The mean plasma level of selenium was below the lower limit of its reference intervals in 61.9% of men and 62.5% of women and above than the upper limit of its reference intervals only in 8.3% of men and 4.2% of women. No correlation between micronutrient intakes and their plasma concentrations was found.

According to age, analysis of plasma concentrations of micronutrients showed no significant difference between men and women in vitamin C, vitamin E, and the VitC/VitE ratio in young and middle-aged adults. In seniors, the plasma concentrations of vitamin C and vitamin E were lower in men than in women (*p* = 0.014 and *p* = 0.042, respectively) (Table 10). In adults, the plasma concentrations of vitamin E were higher in adult men than in women, and the VitC/VitE ratio was lower in men than in women (*p* = 0.009 and *p* = 0.015, respectively). While no difference in the VitE/Chol ratio was observed at all ages, higher plasma cholesterol levels were observed in senior adults (*p* = 0.017). The plasma levels of zinc were only higher in young men than in women (*p* = 0.023). The copper and the Cu/Zn ratio were lower in young, adult, and middle-aged men than in women (copper: *p* = 0.003, *p* < 0.001, and *p* < 0.001; Cu/Zn ratio: *p* = 0.001, *p* < 0.001, and *p* < 0.001, respectively). No significant difference in selenium between men and women was observed at all ages. The mean plasma concentrations of vitamin E, copper, zinc, and VitE/Chol ratio were within their reference intervals in men and women of all ages. Only in men were the plasma concentrations of vitamin C below the lower limit of their reference intervals at all ages. In women, the plasma concentrations of vitamin C were below the lower limit of their reference intervals in young and middle-aged adults. Furthermore, the VitC/VitE ratio was below the lower limit of their reference intervals in men and women at all ages. Cholesterol plasma levels were within their reference intervals only in men of all ages. While in young and adult women, cholesterol plasma levels were within their reference intervals, they were above the upper limit of their reference intervals in middle-aged and senior women. At all ages, the Cu/Zn ratio was below the lower limit of the reference intervals only in men. It was within their reference intervals for senior women and above the upper limit of their reference intervals for the other ages. In young and middle-aged adults, the Cu/Zn ratio was below the lower limit of their reference intervals in women. Finally, in men and women, the plasma concentrations of selenium were below the lower limit of their reference intervals at all ages.

### 3.5. Dietary and Micronutrient Dietary Intakes and Plasma Concentrations in 32 Patients with FSHD

Dietary energy and macronutrients intakes in the subgroup of 32 patients who underwent MRI were similar to those of the whole sample (*n* = 159 patients) (Supplementary Table S2). Conversely, none of the between-sex differences in micronutrient dietary intakes observed in the larger group (*n* = 159) were detected in the subgroup. The mean values of most plasma micronutrient concentrations in patients with FSHD were within the reference intervals (Table 11). However, the VitC/VitE ratio was below the lower limit, and the Cu/Zn ratio, lipid peroxides, and urinary oxidized DNA levels were above the upper limit of their reference intervals (Table 11).

Comparison of the plasma concentrations of these micronutrients in patients with FSHD (*n* = 32) and controls (*n* = 7) showed that plasma vitamin E levels were comparable between groups (*n* = 7), but plasma vitamin C levels were significantly lower in patients with FSHD (*p* < 0.001) (Table 7). Moreover, the VitC/VitE ratio was significantly lower in patients than in healthy controls (*p* = 0.006). Additionally, while the VitE/Chol ratio was similar in patients with FSHD and healthy controls, cholesterol levels were higher in patients than in healthy controls (*p* = 0.033). The plasma concentrations of selenium and copper were similar in patients with FSHD and healthy controls, whereas the zinc concentration was significantly lower and the Cu/Zn ratio was higher in patients than in healthy controls (*p* = 0.022 and *p* = 0.05, respectively).

A comparison of the plasma concentrations between the sexes showed a lower VitE/Chol ratio in women than in men with FSHD (*p* = 0.016). Conversely, the plasma concentrations of copper and the Cu/Zn and VitC/VitE ratios were higher in women than in men with FSHD (*p* < 0.001, *p* = 0.001, and *p* = 0.025, respectively). The plasma levels of cholesterol, zinc, and selenium were similar between sexes.

The mean plasma concentrations of vitamin C and vitamin E were below the lower limit of their reference intervals in 55% and 5% of men and in 25% and 8.3% of women, and above the upper limit of their reference intervals in 5% and 10% of men and in 8.3% and 0% of women, respectively. The VitC/VitE and VitE/Chol ratios were below the lower limit of their reference intervals in 80% and 5% of men and in 50% and 0% of women, respectively, and above the upper limit of their reference intervals in 5% and 35% of men and in 33% and 0% of women, respectively. The mean copper and zinc plasma levels were below the lower limit of their reference intervals in 5% and 20% of men and in 0% and 8.3% of women, respectively, and above the upper limit of their reference intervals in 0% of men and in 16.7% and in 0% of women, respectively. The mean Cu/Zn ratio was below the lower limit of its reference intervals in 30% of men and in 0% of women and above the upper limit of its reference intervals in 15% of men and in 83.3% of women. The mean plasma level of selenium was below the lower limit of its reference intervals in only 30% of men and 25% of women and above than the upper limit of its reference intervals in 20% of men and in 0% of women. No correlation between micronutrient intakes and their plasma concentrations was found.

### 3.6. Antioxidant Status and Oxidative Stress Markers in 32 Patients with FSHD and 7 Healthy Controls

Whole blood glutathione peroxydase (GSH-Px) and copper-zinc dependent superoxide dismutase (Cu/Zn SOD) activities were significantly lower in patients with FSHD than in healthy controls (*p* = 0.05 and *p* < 0.001, respectively) (Table 12). No difference in total GSH pool (reference interval: 717–1110), whole blood reduced GSH (reference interval: 715–1090), oxidized glutathione (GSSG) (reference interval: 0.96–10), and the GSH/GSSG ratio (reference interval: 111–747) was observed in patients with FSHD compared with healthy controls. The plasma lipid peroxides (reference interval < 432) and urinary oxidized DNA (OxDNA) levels (reference interval: 0–16) were significantly higher in patients with FSHD than in healthy controls (*p* < 0.001).

Analysis of antioxidant status markers showed no difference between sexes in whole blood reduced GSH, GSSG and total GSH pool levels, the GSH/GSSG ratio, whole blood GSH-Px, and CuZnSOD activities (Table 12). Similarly, no difference between sexes in urinary oxidized DNA and oxidized DNA/creatinine ratio was observed. Conversely, lipid peroxides were significantly higher in women than in men (*p* < 0.001) (Table 12). The mean values for lipid peroxides were above the upper limit of the reference intervals in 30% of men and in 83.3% of women.

### 3.7. MRI Evaluation of Thigh Parameters in 32 Patients with FSHD and 7 Healthy Controls

The thigh with the highest MVC_Q_ was considered the dominant thigh and the other the non-dominant thigh. Dominant MVC_Q_ was significantly lower in patients with FSHD than in healthy controls (*p* < 0.001) (Table 13). Both dominant and non-dominant quadriceps MVC_Q_ were higher in men (*n* = 20) than in women with FSHD (*n* = 12) (*p* = 0.014 and *p* = 0.069, respectively). More than 300 MRI images per participant were analyzed to investigate quadriceps and post-muscle volumes and fat infiltration [24], and not only the cross-sectional surface area [49,50]. MRI analysis of thigh volumes showed no difference in dominant and non-dominant TV_T_ between patients with FSHD and healthy controls (Table 13). Conversely, significant differences in MV_T,_ FV_T_, %MV_T_, and %FV_T_ were found between patients with FSHD and healthy controls. Specifically, MV_T_ (dominant thigh) was lower (*p* = 0.037) and FV_T_ (dominant and non-dominant thigh) was higher in patients with FSHD than healthy controls (*p* < 0.001, respectively). Conversely, %MV_T_ (dominant and non-dominant thighs) was lower and %FV_T_ was higher (dominant and non-dominant) in patients with FSHD than in healthy controls (*p* < 0.001, respectively).

In patients with FSHD, TV_T_ and MV_T_ (dominant and non-dominant) were higher in men than in women (*p* < 0.001; *p* < 0.001; *p* = 0.040; and *p* = 0.009, respectively). Conversely, FV_T_, %MV_T_, and %FV_T_ (dominant and non-dominant) were not different between men and women with FSHD. Similar results were obtained for between-sex comparisons in healthy controls (Table 13).

A comparison of the dominant and non-dominant thigh volume parameters in patients with FSHD showed no difference in FV_T_, whereas %FV_T_ and TV_T_ were higher in the dominant than in the non-dominant thigh in all patients (*p* = 0.005 and *p* = 0.001, respectively). MV_T_ and % MV_T_ were lower in the dominant than in the non-dominant thigh (*p* = 0.003, *p* = 0.005, respectively). In healthy controls, only MV_T_ and TV_T_ were lower in the dominant than in the non-dominant thigh (*p* = 0.008, *p* = 0.018, respectively). Moreover, volume parameters were similar in the dominant and non-dominant thigh in women with FSHD. Conversely, dominant MV_T_ and %MV_T_ were significantly lower, and %FV_T_ was higher in the dominant than in the non-dominant thigh in men with FSHD (*p* = 0.004 and *p* = 0.048, respectively). In healthy controls, no difference between the dominant and non-dominant thigh was observed in men and women.

### 3.8. Correlations between Energy Dietary Parameters and Micronutrient Intakes in 159 Patients with FSHD

Analysis of the correlations between CI and energy dietary intake showed that CI was positively correlated with protein per kilogram of body weight in all patients and also in men and women (r = 0.316; r = 0.531; and r = 0.415, *p* < 0.001, respectively) (Table 14). CI was negatively correlated with the protein/energy ratio (%) in all patients and also in men and women with FSHD (r = −0.316, *p* < 0.001; r = −0.372, *p* < 0.001; and r = −0.296, *p* = 0.011, respectively). CI was positively correlated with calorie intakes from proteins, lipids, and carbohydrates in all patients and also when they were divided by sex (proteins: r = 0.489, r = 0.494, and r = 0.557, *p* < 0.001; lipids: r = 0.637, r = 0.731, and r = 0.816, *p* < 0.001; carbohydrates: r = 0.776, r = 0.744, and r = 0.776, *p* < 0.001, respectively). Similarly, CI was positively correlated with vitamin E intake in all patients and both in men and women (r = 0.470, r = 0.463, and r = 0.421, *p* < 0.001, respectively) and inversely correlated with the VitC/VitE ratio intake in all patients and in men (r = −0.245, *p* = 0.003 and r = −0.238, *p* = 0.026, respectively). CI was positively correlated with cholesterol intake in all patients and in men and women with FSHD (r = 0.317, *p* < 0.001; r = 0.357, *p* = 0.001; and r = 0.233, *p* = 0.047, respectively). CI was positively correlated with selenium intake in all patients, and in men (r = 0.353, *p* < 0.001 and r = 0.365, *p* = 0.001, respectively). CI was positively correlated with copper and zinc intakes in all patients and in men and women (copper: r = 0.429; r = 0.382, and r = 0.385, *p* < 0.001; zinc: r = 0.492; *p* < 0.001, r = 0.321, *p* = 0.004, and r = 0.581, *p* < 0.001, respectively). A negative correlation between CI and Cu/Zn ratio intake was observed only in women (r = −0.281, *p* = 0.016).

### 3.9. Correlations between Micronutrients Intakes in 159 Patients with FSHD

Copper and zinc intakes were positively correlated in all patients and also in men and women with FSHD (r = 0.350, *p* < 0.001; r = 0.268, *p* = 0.014; and r = 0.379, *p* < 0.001, respectively) (Table 14). The Cu/Zn ratio was positively correlated with copper intake and inversely correlated with zinc intake in all patients and in men and women (r = 0.430, *p* < 0.001; r = 0.519, *p* < 0.001; r = 0.371, *p* = 0.001; r = −0.648, *p* < 0.001; r = −0.641, *p* < 0.001; and r = −0.671, *p* < 0.001, respectively). Copper and zinc were correlated with selenium in all patients and in men and women (r = 0.288, *p* < 0.001; r = 0.252, *p* = 0.021; r = 0.270, *p* = 0.020; r = 0.296, *p* < 0.001; r = 0.271, *p* = 0.013; and r = 0.267, *p* = 0.022, respectively) (Table 14).

### 3.10. Correlations between Age and Micronutrient Intakes and Plasma Concentration in 159 Patients with FSHD

Age was positively correlated with Vitamin C intake in all patients and in men (r = 0.164, *p* = 0.040 and r = 0.282, *p* = 0.010, respectively) (Table 14). A positive correlation between age and VitC/VitE ratio intake was only observed in men (r = 0.265, *p* = 0.015). Additionally, the positive correlation between age and plasma vitamin E concentration in all patients was maintained in men and women (r = 0.358, *p* < 0.001; r = 0.324, *p* = 0.003; and r = 0.357, *p* = 0.002, respectively) (Table 14). Moreover, a negative correlation between age and plasma vitamin C concentration was found in men (r = −0.233, *p* = 0.035). The negative correlation between age and the VitC/VitE ratio in all patients was only maintained in men (r = −0.266, *p* = 0.001 and r = −0.338, *p* = 0.002, respectively). Finally, the positive correlation between age and plasma cholesterol levels in all patients was maintained in men and women (r = 0.469, *p* < 0.001; r = 0.380, *p* < 0.001; and r = 0.551, *p* < 0.001, respectively).

### 3.11. Correlations between PAL and Energy Dietary Parameters and Micronutrient Intakes and Plasma Concentration in 159 Patients with FSHD

PAL was positively correlated with CI in all patients (r = 0.302, *p* = 0.001) and also with CI from proteins and carbohydrates in all patients (r = 0.215, *p* = 0.019 and r = 0.271, *p* = 0.003, respectively). (Table 14). PAL was also positively correlated with copper and zinc intakes in all patients and in men (r = 0.195, *p* = 0.033; r = 0.306, *p* = 0.016; r = 0.270, *p* = 0.003; and r = 0.282, *p* = 0.027, respectively), as well as with selenium intake in all patients and in men (r = 0.204, *p* = 0.026, respectively). Conversely, PAL was negatively correlated with plasma copper in all patients and positively correlated in women (r = −0.355, *p* < 0.001 and r = 0.281, *p* = 0.034, respectively). PAL was negatively correlated with the plasma Cu/Zn ratio in all patients (r = −0.351, *p* < 0.001).

### 3.12. Correlations between Energy Dietary Parameters and Micronutrient Intakes and Plasma Concentration and Oxidative Stress Markers in 32 Patients with FSHD

All correlations between dietary energy parameters in the larger patient group (*n* = 159) and the men in this group were also detected in the subgroup of 32 patients and the men in this subgroup (Table 15). However, some of the correlations observed in women from the larger group were not found in the women in the subgroup. This concerned the correlations between CI and protein/energy ratio (%) and calorie intakes from proteins. Moreover, some correlations between energy dietary parameters and micronutrient intakes in the group of 159 patients were not maintained in the subgroup of 32 patients. This concerned the correlations between CI and selenium intake and the correlations between CI and the Cu/Zn and VitC/VitE ratios. Finally, analysis of the correlations between CI and oxidative stress markers showed that only CI was negatively correlated with the oxidized DNA/creatinine ratio in women (r = −0.645, *p* = 0.029).

### 3.13. Correlations between Dietary Energy and Muscle Parameters in 32 Patients with FSHD

CI and dominant and non-dominant MV_T_ were positively correlated in all patients with FSHD and in women (dominant thigh: r = 0.396, *p* = 0.027; r = 0.718, *p* = 0.011 and non-dominant thigh: r = 0.349, *p* = 0.05; r = 0.655, *p* = 0.026, respectively) (Table 15). Similarly, CI and dominant and non-dominant %MV_T_ were positively correlated in all patients with FSHD and in women (dominant thigh: r = 0.384, *p* = 0.033; r = 0.800, *p* = 0.001 and non-dominant thigh: r = 0.347, *p* = 0.050; r = 0.745, *p* = 0.007, respectively). CI was negatively correlated with dominant and non-dominant FV_T_ and %FV_T_ in all patients with FSHD and in women (dominant FV_T_: r = −0.800, *p* = 0.001; non-dominant FV_T_: r = −0.764, *p* = 0.050; and dominant %FV: r = −0.384, *p* = 0.032; r = −0.800, *p* = 0.001; non-dominant %FV: r = −0.347 *p* = 0.05; r = −0.745, *p* = 0.007). Moreover, CI was positively correlated with dominant and non-dominant TV_T_ only in the whole sample (r = 0.369, *p* = 0.041 and r = 0.352, *p* = 0.05, respectively) (Table 15). PAL was positively correlated with CI in all patients and in women (r = 0.415, *p* = 0.020 and r = 0.671, *p* = 0.021, respectively) (Table 15). PAL was negatively correlated with protein/energy% only in women and negatively correlated with lipid/energy% only in men (r = −0.671, 0.021, and r = −0.495, *p* = 0.026). The correlations between PAL and protein (kcal) and carbohydrate (kcal) intakes observed in the group of 159 patients were maintained in the subgroup of 32 patients (r = 0.434, *p* = 0.015 and r = 0.388, *p* = 0.031). PAL was also correlated with carbohydrate (kcal) intakes in women in the subgroup (r = 0.671, *p* = 0.021) (Table 15).

### 3.14. Correlations between Muscle Parameters and Plasma Concentrations of Micronutrients and Oxidative Stress Markers in 32 Patients with FSHD

Analysis revealed no correlations between muscle parameters and systemic antioxidant status/oxidative stress markers in healthy controls, as previously reported [21].

Vitamin E was negatively correlated with dominant FV_T_ and %FV_T_ only in women (r = −0.664, *p* = 0.017 and r = −0.629, *p* = 0.026) (Table 15) and positively correlated with dominant %MV_T_ only in women (r = 0.629, *p* = 0.026). In all patients, VitC/VitE ratio was negatively correlated with dominant and non-dominant TV_T_ (r = −0.439, *p* = 0.012 and r = −0.467, *p* = 0.007). VitE/chol was positively correlated with non-dominant MV_T_ only in women (r = 0.657, *p* = 0.019) and with non-dominant TV_T_ in all patients (r = 0.414, *p* = 0.019). VitE/chol was also positively correlated with dominant and non-dominant TV_T_ (r = 0.629, *p* = 0.026 and r = 0.776, *p* = 0.002) in women. Copper was positively correlated with dominant MV_T_ (r = 0.615, *p* = 0.031) only in women, and the Cu/Zn ratio was positively correlated with dominant %MV_T_ only in men (r = 0.450, *p* = 0.046).

Total GSH pool and whole blood reduced GSH levels were positively correlated with dominant %MV_T_ and inversely correlated with dominant %FV_T_ only in men (r = 0.445, *p* = 0.048; r = 0.447, *p* = 0.048; r = −0.445, *p* = 0.048; and r = −0.447, *p* = 0.048, respectively) (Table 15). Blood CuZnSOD activity was positively correlated with dominant and non-dominant MV_T_ and inversely correlated with FV_T_ only in women (r = 0.661, *p* = 0.033; r = 0.697, *p* = 0.022; r = −0.697, *p* = 0.022; and r = −0.673, *p* = 0.029, respectively). Blood CuZnSOD activity was positively correlated with dominant thigh TV_T_ women (r = 0.648, *p* = 0.038). Moreover, blood CuZnSOD activity was positively correlated with dominant and non-dominant %MV_T_ and inversely correlated with %FV_T_ only in women (r = 0.733, *p* = 0.013; r = 0.673, *p* = 0.029; r = −0.733, *p* = 0.013; and r = −0.673, *p* = 0.029, respectively). Lipid peroxides were negatively correlated with both dominant and non-dominant MV_T_ in all patients and in men (r = −0.427, *p* = 0.017 vs. r = −0.471, *p* = 0.007; r = −0.498, *p* = 0.025; and r = −0.540, *p* = 0.014, respectively). Similarly, lipid peroxides were negatively correlated with both dominant and non-dominant TV_T_ in all patients and in men (r = −0.584, *p* < 0.001; r = −0.615, *p* < 0.001; r = −0.567, *p* = 0.009; and r = −0.519, *p* = 0.019, respectively). Furthermore, a positive correlation between lipid peroxides and non-dominant FV_T_ was observed only in men with FSHD (r = 0.463, *p* = 0.039). Lipid peroxides were correlated negatively with dominant and non-dominant %MV_T_ and positively with dominant and non-dominant %FV_T_ only in men (%MV_T_: r = −0.450, *p* = 0.046; r = −0.535, *p* = 0.015; %FV_T_: r = 0.450, *p* = 0.046; and r = 0.535, *p* = 0.015, respectively). Oxidized DNA/creatinine ratio was negatively correlated with dominant and non-dominant MV_T_ and %MV_T_ in all patients and men (MV_T_: r = −0.635, *p* < 0.001; r = −0.566, *p* < 0.001; r = −0.725, *p* < 0.001; and r = −0.674, *p* = 0.001 and %MV_T_: r = −0.637, *p* < 0.001; r = −0.584, *p* < 0.001; r = −0.735, *p* < 0.001; and r = −0.642, *p* = 0.003, respectively). Conversely, the oxidized DNA/creatinine ratio was positively correlated with dominant and non-dominant FV_T_ and %FV_T_ in all patients and in men (FV_T_: r = 0.633, *p* < 0.001; r = 0.585, *p* < 0.001; r = 0.682, *p* = 0.001; and r = 0.654, *p* = 0.002 and %FV_T_: r = 0.637, *p* < 0.001; r = 0.584, *p* < 0.001; r = 0.735, *p* < 0.001; and r = 0.642, *p* = 0.003, respectively). The oxidized DNA/creatinine ratio was negatively correlated with dominant TV_T_ in all patients and in men (r = −0.362, *p* = 0.045 and r = −0.532, *p* = 0.019, respectively), and with non-dominant TV_T_ only in men (r = −0.474, *p* = 0.039) (Table 15). Finally, the plasma Cu/Zn ratio was negatively correlated with PAL in the group of 159 patients and also in the subgroup of 32 patients (r = −0.387, *p* = 0.032) (Table 15).

### 3.15. Correlations between PAL and Muscle Parameters in 32 Patients with FSHD

PAL was positively correlated with non-dominant TV_T_ in all patients (r = 0.365, *p* = 0.040) (Table 15). PAL was negatively correlated with dominant and non-dominant FV_T_ in men (r = −0.481, *p* = 0.031 and r = −0.458, *p* = 0.042) and with dominant and non-dominant %FV_T_ in men (r = −0.435, *p* = 0.05 and r = −0.461, *p* = 0.040). PAL was positively correlated with dominant and non-dominant %MV_T_ in men (r = 0.435, *p* = 0.05 and r = 0.461, *p* = 0.040).

### 3.16. Correlations between Plasma Concentrations of Micronutrients and Oxidative Stress Markers in 32 Patients

Plasma copper level was positively correlated with the Cu/Zn ratio in all patients and only in women (r = 0.641, *p* < 0.001 and r = 0.741, *p* = 0.005) (Table 15). Plasma vitamin C concentration was positively correlated with plasma copper level and Cu/Zn ratio in all patients (r = 0.411, *p* = 0.019 and r = 0.445, *p* = 0.012, respectively). Plasma copper level and Cu/Zn ratio were correlated with lipid peroxides in all patients and in women (r = 0.729, *p* < 0.001; r = 0.502, *p* = 0.005; r = 0.936; and r = 0.855, *p* < 0.001). Moreover, cholesterol level was positively correlated with plasma vitamin E concentration in all patients and in men (r = 0.588, *p* < 0.001 and r = 0.637, *p* = 0.020, respectively). Plasma vitamin E concentration was negatively correlated and plasma vitamin C concentration was positively correlated with the VitC/VitE ratio in all patients and in men and women (ALL: r = −0.742, *p* < 0.001; r = 0.91, *p* < 0.001; men: r = −0.674, *p* = 0.001; r = 0.732, *p* < 0.001; and women: r = −0.692, *p* = 0.011; r = 0.895, *p* < 0.001, respectively). The VitC/VitE ratio and cholesterol level were negatively correlated only in the whole sample (r = −0.422, *p* = 0.016). The VitC/VitE ratio was positively correlated with plasma copper level and lipid peroxides only in the whole sample (r = 0.376, *p* = 0.034 and r = 0.433, *p* = 0.015, respectively). Total GSH pool level was positively correlated with whole blood reduced GSH levels in all patients and also in men and women (r = 0.991; r = 0.988; and r = 0.993, *p* < 0.001, respectively). Total GSH pool level was also positively correlated with GSSG level and inversely correlated with the GSH/GSSG ratio in women (r = 0.741; *p* = 0.005 and r = −0.657; *p* = 0.019, respectively). Whole blood reduced GSH level was positively correlated with the GSH/GSSG ratio in men and inversely correlated in women (r = 0.454, *p* = 0.043 and r = −0.622, *p* = 0.028, respectively). GSSG was positively correlated with whole blood reduced GSH level only in women (r = 0.720, *p* = 0.007) and negatively correlated with the GSH/GSSG ratio in all patients and also in men and women (r = −0.985; r = −0.976; and r = −0.972, *p* < 0.001, respectively). Copper level was positively correlated with the GSH/GSSG ratio and inversely correlated with GSSG level in men (r = 0.655, *p* = 0.002 and r = −0.653, *p* = 0.002, respectively). The oxidized DNA/creat ratio was negatively correlated with total GSH pool level and inversely correlated with the Cu/Zn ratio only in men (r = −0.486, *p* = 0.034 and r = 0.561, *p* = 0.014, respectively). The oxidized DNAox/creat ratio was negatively correlated with whole blood reduced GSH level in all patients and in men (r = −0.373, *p* = 0.039 and r = −0.479, *p* = 0.037, respectively). Reduced GSH was positively correlated with lipid peroxides only in women (r = 0.618, *p* = 0.039).

## 4. Discussion

FSHD is characterized by progressive deterioration of muscle mass, strength, and function and affects both sexes equally. Previous studies have highlighted relationships between some neuromuscular diseases and nutrition [45,51,52,53]. Data on the eating habits and nutrition of patients with FSHD are extremely limited [54]. To our knowledge, this is the first study on a large population of FSHD patients that investigated dietary intake with a focus on sex differences. One of our main findings is that the overall energy intake was lower in patients with FSHD, corroborated by the results obtained by Motlagh et al. [54]. In this present study, 84% and 53% of patients with FSHD did not meet the RNI for daily energy intake and for protein intake, respectively. The positive correlations between daily energy intake and protein per kilogram of body weight and protein kilocalories in all patients suggest that low protein intake may be a major contributor to the relatively low energy intake in patients with FSHD. Daily energy intake was positively correlated with physical activity level. Low levels of physical activity (evaluated using the self-reported Voorrips physical activity questionnaire [55]) concerned 84% of patients. Moreover, 38% and 6% of patients with FSHD had high intakes of fat and carbohydrates, respectively. Among our patients, 25% and 7% were categorized as overweight (25 ≤ BMI < 29.9) and obese (BMI < 30), respectively. Overweight and obesity are common in patients with FSHD. Physical inactivity due to muscle weakness has been widely cited as a contributor to obesity in patients with FSHD [56]. In addition to other contributing lifestyle factors, a high intake of carbohydrates and fat should also be considered as contributing to overweight and obesity in these patients. Furthermore, 13% of patients had BMI values <18.5 (i.e., underweight category).

The prevalence of inadequate micronutrient intakes was generally low, notably for zinc, vitamin C, vitamin E, and the vitamin E/cholesterol ratio. Zinc intake was positively correlated with physical activity level and was inadequate in 85% of patients. Although this correlation does not prove cause and effect, suboptimal zinc intake may influence physical activity. In this study, we also determined whether their plasma concentrations were reduced. Most of the plasma concentration of nutrients were within their reference intervals. However, the plasma concentrations of vitamin C, selenium, and the Cu/Zn and VitC/VitE ratios were below the lower limit of their reference intervals in 53.2%, 62.2%, 39.1%, and 73.3% of patients, respectively.

Our study also highlighted sex-related disparities in energy and macronutrient intakes. Intake of dietary energy were higher in men than in women. However, according to age, adult men had a higher calorie intake than women. Yet, the daily dietary intakes were inadequate in most men except for seniors. Intake of macronutrients was also higher in men than in women in the entire population. The mean protein/fat and carbohydrate/energy ratios were not different between women and men. While no significant difference was observed in the percentage of carbohydrates, proteins, and lipids between men and women, the mean kilocalories from proteins and carbohydrates were significantly higher in men than in women. Additionally, differences in the intake for all 3 classes of macronutrients (proteins, fat, and carbohydrates) between men and women were only observed in adults and in middle-aged adults for carbohydrates. Moreover, men and women did not meet their RNI for various minerals. The frequency of inadequate intake (both sexes and at all ages) was higher for vitamin C, vitamin E, and zinc.

Vitamin E, zinc, and copper intakes were lower in women than in men in the entire population. Zinc and copper intakes were insufficient in 87% and 61% of men and 82% and 7% of women, respectively. A positive correlation between zinc and copper intakes was observed in both sexes. However, physical activity level was positively correlated with zinc and copper intakes only in men, suggesting that in women, their lower intakes may not be related to physical activity level. According to age, we observed lower vitamin E, zinc, and copper intakes in adult women than in men, suggesting a change in dieting behavior in adulthood between men and women. With age, the differences in mean PAL between men and women peaked in middle-aged adults and declined thereafter.

Vitamin E levels were within the reference range but not adequate to counteract DNA damage. As the vitamin C mean value was in the lower limit of the reference range, vitamin C may not efficiently complement and potentiate vitamin E antioxidant activity [57], which ensures maximum protection against oxidative damage. Moreover, in the present study, no difference in zinc plasma levels was observed between sexes, and they were within their reference intervals in most patients. Conversely, copper plasma levels and the copper/zinc ratio were higher in women than in men. No correlations between the zinc dietary intake and plasma copper levels and the copper/zinc ratio were observed. As previously described [22,23,24], the levels of oxidative stress markers and antioxidant molecules (GSSG, GSH-Px, CuZnSOD, lipid peroxides, and oxidized DNA) were significantly higher in patients with FSHD than in healthy controls.

Systemic inflammation negatively affects zinc and copper concentrations in serum, resulting in an increased copper/zinc ratio [58]. Elevated copper/zinc ratios have been associated with malnutrition, increased oxidative stress, inflammation, and disrupted immune status in patients with chronic diseases [59]. As previously reported, the significant positive correlation between lipid peroxides and both copper and the copper/zinc ratio in patients with FSHD was probably explained by the higher mean copper (but not zinc) levels, mean copper/zinc ratios, and lipid peroxide levels (both above the upper limit of the reference ranges) in women than in men. Interestingly, while none of the between-sex differences micronutrient dietary intakes observed in the larger group were detected in the subgroup, between-sex differences in plasma copper and Cu/Zn and VitC/VitE ratios are maintained. Moreover, thigh muscle volume parameters were analyzed by MRI in 32 patients with FSHD and 7 healthy controls. The total volume of dominant and non-dominant thighs was not different between patients with FSHD and controls. However, fat and fat percentage volumes of both thighs were higher in the patients than in the controls, as previously reported [24]. Only the muscle volume of the dominant thigh was lower in patients than in healthy controls. Moreover, comparison of the muscle volume parameters between dominant and non-dominant thighs indicated that muscle volume was higher in the dominant thigh than in the non-dominant thigh in both patients and healthy controls. Additionally, as expected, differences in thigh muscle volumes were observed between women and men (patients and controls); total and muscle volumes of both thighs were higher in men than in women. However, no sex-related difference in fat and fat percentage volumes of both thighs was found in patients and healthy controls. On the other hand, in patients, differences between dominant and non-dominant thigh muscle volume parameters were observed only in men. Specifically, muscle volume was lower in the dominant thigh, and fat percentage was higher in the dominant thigh than in the non-dominant thigh in men. Analysis of the correlations between muscle volume parameters and energy dietary intake showed that all muscle volume parameters (both thighs) were correlated with dietary energy intake, suggesting that inadequate dietary energy intake may be related to the progressive loss of muscle mass in patients with FSHD. Muscle volume parameters were correlated with energy dietary intake only in women, suggesting that the more important dietary energy deficiency in women may contribute to their higher loss of muscle mass compared with men. Additionally, significant correlations between oxidative stress markers and muscle parameters were observed. In men, plasma levels of lipid peroxides and urinary levels of oxidized DNA/creatinine ratio were negatively correlated with thigh muscle volume and total muscle volume, suggesting that the increased lipid peroxides and oxidized DNA may contribute to muscle loss.

A low energy intake is a risk factor for suboptimal intake of proteins, vitamins, and minerals that are important for protein synthesis and other metabolic pathways and that might contribute to the progressive loss of muscle mass. Most patients with muscle dystrophy, such as myotonic muscular dystrophy type 1, and patients with FSHD do not meet their daily energy requirements [54]. This might promote the oxidative stress and mitochondrial dysfunction and inflammation that is observed in patients with FSHD. Strategies to increase energy and protein intakes will also contribute to increasing the intake of vitamins and minerals. However, given the extent of the observed deficits, supplementation of some key micronutrients and/or vitamins could be beneficial for patients. This hypothesis is supported by the absence of a correlation between dietary energy and nutrient intakes. Accordingly, in a previous randomized clinical trial on 53 patients with FSHD (NCT01596803), we evaluated the impact on physical muscle performance of oral administration of vitamin C (ascorbic acid, 500 mg), vitamin E (a-tocopherol, 400 mg), L-selenomethionine (200 µg), and zinc gluconate (25 mg) [23]. Our results suggest that the antioxidant response can be improved by antioxidant supplementation that reduces oxidative stress and increases the antioxidant defenses associated with muscle strength [23]. Moreover, the baseline levels of all anti-oxidants included in this supplementation could predict the treatment response, suggesting that they are all required for the treatment efficacy. If only women were considered, no significant difference in the physical performance values was found in both supplemented and placebo groups between baseline and treatment end. This suggests that in women with high basal plasma concentrations of copper and a high copper/zinc ratio, supplementation does not have any significant effect on physical performance [23].

Our study has some limitations. The nutritional and dietary intakes were obtained through the analysis of a dietary diary kept by the participants. As the types and quantities of foods and beverages listed in the diary were self-reported, they may not be representative of what they really consumed due to a lack of interest or a lack of understanding of how to correctly fill in the diary. Moreover, our study did not include a control group for dietary intake analysis. Therefore, it was impossible to assess whether our results are representative of the nutritional and dietary intakes of women and men with FSHD.

## 5. Conclusions

In patients with FSHD, the mean daily CI is lower in women than in men. However, according to age, it was significantly lower only in adult women than in men. The mean zinc, vitamin C, and vitamin E dietary intakes were below the RNI recommendations in most patients (both sexes). The plasma concentrations of vitamin C, selenium, and the Cu/Zn and VitC/VitE ratios were below the lower limit of their reference intervals in most patients. No correlation between nutrient intakes and their plasma concentration was found. A varied diet generally provides enough micronutrients. However, patients with FSHD may need supplements to correct vitamin/mineral deficiencies.

## Figures and Tables

**Table 1:** Demographic, clinical, and anthropometric characteristics of patients with FSHD. SD, standard deviation; min, minimum; max, maximum; BMI, body mass index.

	All Patients (*n* = 159)	Men (*n* = 85)		Women (*n* = 74)		
Parameters	Mean ± SD	Mean ± SD	Median (Min–Max)	Mean ± SD	Median (Min–Max)	*p* Value
Age (years)	42.7 ± 14.9	43.7 ± 14.7	45 (14.0–75.0)	41.7 ± 14.5	41 (14.0–69.0)	0.399
D4Z4 (repeat units)	5.6 ± 1.9	5.7 ± 1.8	6.0 (1.0–9)	5.6 ± 1.8	5.0 (2.0–9.0)	0.772
Body weight (kg)	68.8 ± 16.4	74.9 ± 13.1	73.5 (45.5–124.4)	61.9 ± 13.2	62.8 (35.6–95.8)	<0.001
Body height (cm)	171.5 ± 21.4	177.3 ± 8.2	176.5 (153.0–195.0)	164.9 ± 6.4	165.0 (144.0–180.0)	<0.001
BMI (kg/m^2^)	23.3 ± 4.96	23.8 ± 3.7	23.9 (15.6–36.3)	22.8 ± 4.8	21.8 (13.1–37.4)	0.132
Waist circumference (cm)	85 ± 17.8	90.2 ± 14.7	89 (65.0–143.0)	79.2 ± 13.4	75.3 (57.0–111.0)	<0.001
Hip circumference (cm)	97.3 ± 14	97.8 ± 7.9	96.3 (80.0–123.0)	96.7 ± 10	95.5 (77.0–120.0)	0.418
Waist/Hip ratio	0.87 ± 0.14	0.92 ± 0.11	0.89 (0.73–1.22)	0.82 ± 0.08	0.81 (0.68–1.08)	<0.001

**Table 2:** Demographic and anthropometric characteristics of patients with FSHD according to age. SD, standard deviation; min, minimum; max, maximum; BMI, body mass index.

		Men	Women	
Parameters	Ages (Years)	Mean ± SD	Mean ± SD	*p* Value
Age (years)	14–30	22.8 ± 4.9	22.8 ± 4.9	0.979
	31–50	41.8 ± 5.8	40.5 ± 5.6	0.352
	51–64	56.3 ± 4.2	58.2 ± 5.0	0.217
	65–75	70.3 ± 3.4	67.2 ± 1.3	0.062
Body weight (kg)	14–30	68.8 ± 11.8	55.7 ± 10.2	0.001
	31–50	76.5 ± 12.7	63.0 ± 13.5	<0.001
	51–64	78.4 ± 14.4	67.3 ± 13.3	0.028
	65–75	69.9 ± 6.2	61.9 ± 15.5	0.268
Body height (cm)	14–30	180.1 ± 7.7	166.1 ± 5.7	<0.001
	31–50	179.2 ± 8.5	165.2 ± 7.0	<0.001
	51–64	174.5 ± 5.4	164.5 ± 6.5	<0.001
	65–75	167.7 ± 9.0	161.2 ± 2.5	0.118
BMI (kg/m^2^)	14–30	21.2 ± 3.1	20.2 ± 3.6	0.409
	31–50	23.8 ± 3.4	23.1 ± 4.9	0.488
	51–64	25.7 ± 3.9	24.8 ± 4.1	0.514
	65–75	24.9 ± 1.5	23.9 ± 6.1	0.714
Waist circumference (cm)	14–30	77.0 ± 7.5	70.3 ± 8.7	0.019
	31–50	89.7 ± 11.2	80.2 ± 12.7	0.001
	51–64	98.7 ± 17.0	85.9 ± 14.1	0.028
	65–75	100.7 ± 12.4	83.3 ± 16.9	0.071
Hip circumference (cm)	14–30	93.3 ± 7.0	91.3 ± 9.2	0.477
	31–50	99.0 ± 7.9	97.7 ± 9.2	0.522
	51–64	98.8 ± 8.0	100.8 ± 10.0	0.532
	65–75	100.3 ± 6.8	96.3 ± 13.0	0.518
Waist/Hip ratio	14–30	0.8 ± 0.0	0.8 ± 0.0	0.001
	31–50	0.9 ± 0.1	0.8 ± 0.1	<0.001
	51–64	1.0 ± 0.1	0.8 ± 0.1	<0.001
	65–75	1.0 ± 0.1	0.9 ± 0.1	0.020

**Table 3:** Energy and macronutrient energy intake and intake adequacy analysis in patients with FSHD.

	All Patients	Men (*n* = 85)		Women (*n* = 74)			Men			Women		
Parameters/Recommendation	Mean ± SD	Mean ± SD	Median (Min–Max)	Mean ± SD	Median (Min–Max)	*p* Value	Adequate *n* (%)	Inadequate *n* (%)	Excessive *n* (%)	Adequate *n* (%)	Inadequate *n* (%)	Excessive *n* (%)
Calorie intake (CI)(kcal) Men: 2470–2730; Women: 1995–2205	1783.3 ± 478.2	1914.4 ± 453.6	1816.8 (1142.4–3037.8)	1652.3 ± 342.6	1619.1 (964.8–2568.0)	<0.001	9 (10.7)	71 (84.5)	4 (4.8)	6 (8.1)	62 (83.8)	6 (8.1)
Protein/energy ratio (%) 10–20	19.2 ± 4.6	19.2 ± 4.2	19.1 (9.5–31.6)	19.3 ± 4.1	19.04 (9.3–29.6)	0.800	43 (51.2)	1 (1.2)	40 (47.6)	44 (58.5)	1 (1.4)	29 (39.2)
Protein intake (g/kg body weight/day): 1.2–2	1.24 ± 0.42	1.18 ± 0.39	1.12 (0.42–2.94)	1.29 ± 0.45	1.23 (0.52–3.12)	0.114	33 (39.3)	49 (58.3)	2 (2.4)	36 (48.7)	34 (45.9)	4 (5.4)
Fat/energy ratio (%) 35–40	37.9 ± 7.4	37.4 ± 6.0	37.0 (22.7–55.2)	38.6 ± 6.3	38.6 (20.3–53.5)	0.241	24 (28.6)	31 (36.9)	29 (34.5)	21 (28.4)	22 (29.7)	31 (41.9)
Carbohydrate/energy ratio (%) 40–55	42.8 ± 8.83	43.4 ± 7.5	43.2 (26.2–59.2)	42.1 ± 7.5	42.5 (23.5–70.4)	0.269	50 (59.5)	27 (32.1)	7 (8.3)	45 (60.8)	26 (35.1)	3 (4.0)
Proteins (Kcal)	3245 ± 92.6	342.6 ± 89.2	335.9 (143.9–600.4)	304.0 ± 77.2	299.6 (117–564)	0.004						
Lipids (Kcal)	656.5 ± 199.3	683.2 ± 191.9	660.9 (267.9–1131.8)	626.3 ± 176.7	610.9 (283.4–1127.6)	0.050						
Carbohydrates (Kcal)	739.5 ± 249.6	797.0 ± 257.3	738.5 (395.3–1574.6)	674.2 ± 193.6	683.5 (329.5–1174.3)	0.001						
Physical activity level (PAL)	1.61 ± 0.23	1.66 ± 0.16	1.57 (1.57–2.1)	1.57 ± 0.02	1.56 (1.56–1.64)	<0.001						

**Table 4:** Mean intake of energy and nutrients according to the body mass index of men and women with FSHD.

	Lean BMI < 18.5 kg/m^2^		*p* Value	Normal 18.5 ≤ BMI < 25kg/m^2^		*p* Value	Overweight 25 ≤ BMI < 29.9 kg/m^2^		*p* Value	Obese 29.9 ≤ BMI < 40 kg/m^2^		*p* Value
	Men	Women		Men	Women		Men	Women		Men	Women	
*n* (%)	6 (7.1)	14 (18.9)		48 (57.1)	39 (52.7)		26 (31)	14 (18.9)		4 (4.8)	7 (9.5)	
Age (years)	27.8 ± 12.4	33.4 ± 16.2	0.417	42.1 ± 14.4	42.6 ± 12.7	0.843	48.7 ± 13.7	41.1 ± 15.8	0.139	54.5 ± 4.7	54.6 ± 9.4	0.987
Height (cm)	177.2 ± 10.9	164.9 ± 5.2	0.038	178.1 ± 7.8	165.4 ± 7.7	<0.001	175.7 ± 8.7	163.9 ± 3.7	<0.001	178.0 ± 8.8	164.6 ± 5	0.046
Body weight (kg)	53.7 ± 7.9	46.1 ± 5.3	0.065	70.6 ± 6.8	59.2 ± 6.9	<0.001	83.4 ± 9.7	72.5 ± 3.9	<0.001	102.9 ± 15.7	88.2 ± 6.1	0.158
BMI (kg/m^2^)	17.0 ± 0.9	16.9 ± 1.6	0.906	22.3 ± 1.7	21.6 ± 1.8	0.101	26.9 ± 1.3	26.9 ± 0.9	0.901	32.3 ± 2.7	32.6 ± 2.4	0.868
Calorie intake (Kcal/day)	2005.7 ± 530.7	1694.8 ± 296.4	0.223	1925.6 ± 464.9	1653.2 ± 400.7	0.004	1866.7 ± 433.7	1611.8 ± 380.9	0.064	1699.6 ± 468.6	1519.7 ± 275.8	0.519
Protein/energy ratio (%)	19.4 ± 4.5	19.0 ± 4.0	0.853	18.7 ± 4.3	19.6 ± 4.3	0.325	19.7 ± 4.3	18.3 ± 4.3	0.353	20.2 ± 4.1	20.1 ± 3.3	0.964
Fat/energy ratio (%)	36.2 ± 3.6	36.6 ± 6.0	0.846	37.6 ± 6.6	38.9 ± 6.2	0.309	37.6 ± 5.9	42.0 ± 5.8	0.029	37.2 ± 5.1	33.7 ± 3.9	0.285
Carbohydrate/ energy ratio (%)	44.4 ± 7.2	44.4 ± 6.5	0.994	43.7 ± 7.8	41.4 ± 8.3	0.187	42.8 ± 7.5	39.6 ± 6.8	0.195	42.6 ± 5.9	46.3 ± 2.4	0.314

**Table 5:** Energy and macronutrient energy intake, intake adequacy analysis, and PAL in patients with FSHD categorized by age.

		Men	Women			Men			Women	
Parameters/Recommendation	Ages (Years)	Mean ± SD	Mean ± SD	*p* Value	Adequate	Inadequate	Excess	Adequate	Inadequate	Excess
Calorie intake (kcal) Men: 2400–2730 Women: 1800–2000	14–30	1778.6 ± 456.5	1614.2 ± 277.8	0.210		17 (94.4)	1 (5.6)	3 (17.6)	12 (70.6)	2 (11.8)
Men: 2600–2800 Women: 1995–2205	31–50	1962.0 ± 406.6	1654.5 ± 367.0	0.001	4 (10.5)	34 (89.5)		3 (7.9)	32 (84.2)	3 (7.9)
Men: 2400–2600 Women: 1928–2057	51–64	1823.2 ± 479.7	1573.6 ± 460.3	0.138	2 (8.7)	20 (87)	1 (4.3)		11 (84.6)	2 (15.4)
Men: 2200–2400 Women: 1600–1805	65–75	1833.6 ± 352.8	1772.7 ± 401.6	0.786	3 (50)	1 (16.7)	2 (33.3)	1 (16.7)	3 (60)	2 (33.3)
Protein/energy ratio (%) 15–20	14–30	19.2 ± 4.0	17.7 ± 4.0	0.273	5 (27.8)	3 (16.7)	10 (55.6)	8 (47.1)	5 (29.4)	4 23.5)
10–20	31–50	19.6 ± 4.5	19.7 ± 4.1	0.931	21 (55.3)		17 (44.7)	22 (57.9)		16 (42.1)
10–20	51–64	18.6 ± 4.3	20.5 ± 4.0	0.202	7 (30.4)	4 (17.4)	12 (52.2)	5 (38.5)		8 (61.5)
15–20	65–75	18.2 ± 2.2	18.9 ± 4.3	0.724	3 (50)	1 (16.7)	2 (33.3)	4 (66.7)	1 (16.7)	1 (16.7)
Protein intake (g/kg body weight/day): 1.2–2	14–30	1.3 ± 0.5	1.3 ± 0.4	0.812	7 (38.9)	10 (55.6)	1 (5.6)	2 (11.8)	13 (76.5)	2 (11.8)
0.83–2	31–50	1.2 ± 0.4	1.3 ± 0.5	0.539	32 (84.2)	5 (13.2)	1 (2.6)	33 (86.9)	4 (10.5)	1 (2.6)
0.83–2.2	51–64	1.0 ± 0.3	1.2 ± 0.5	0.184	20 (87)	3 (13)		9 (69.2)	4 (30.8)	0
1–2.2	65–75	1.1 ± 0.2	1.3 ± 0.5	0.321	3 (60)	2 (40)		4 (66.7)	2 (33.3)	0
Fat/energy ratio (%) 35–40	14–30	38.0 ± 5.7	36.5 ± 6.5	0.484	5 (27.8)	7 (38.9)	6 (33.3)	3 (23.1)	7 (53.8)	3 (23.1)
35–40	31–50	37.6 ± 6.0	38.9 ± 6.2	0.346	7 (18.4)	14 (36.8)	17 (44.7)	13 (35.1)	11 (29.7)	13 (35.1)
35–40	51–64	36.3 ± 4.7	39.0 ± 5.9	0.130	11 (47.8)	8 (34.8)	4 (17.4)	4 (36.4)	3 (27.3)	4 (36.4)
35–40	65–75	39.6 ± 11.1	41.6 ± 6.7	0.723	1 (16.7)	2 (33.3)	3 (50)	1 (33.3)	1 (33.3)	1 (33.3)
Carbohydrate/energy ratio (%) 45–50	14–30	42.8 ± 7.1	45.8 ± 8.2	0.066	5 (27.8)	11 (61.1)	2 (11.1)	5 (29.4)	8 (47.1)	4 (23.5)
45–50	31–50	42.8 ± 7.9	41.4 ± 7.1	0.004	9 (39.1)	8 (34.8)	6 (26.1)	8 (21.1)	26 (38.4)	4 (10.5)
40–65	51–64	45.2 ± 6.5	40.5 ± 7.5	0.830	18 (78.3)	5 (21.7)		9 (69.2)	4 (30.8)	
40–65	65–75	42.1 ± 10.0	39.5 ± 5.0	0.888	3 (50)	3 (50)		3 (50)	3 (50)	
Proteins (Kcal)	14–30	330.4 ± 96.1	277.5 ± 63.9	0.066						
	31–50	368.9 ± 94.4	311.6 ± 69.7	0.004						
	51–64	319.5 ± 75.6	312.8 ± 108.3	0.830						
	65–75	306.2 ± 39.5	311.6 ± 82.5	0.888						
Lipids (Kcal)	14–30	654.9 ± 142.5	593.3 ± 141.7	0.209						
	31–50	723.5 ± 210.4	630.5 ± 174.8	0.041						
	51–64	642.9 ± 193.8	616.8 ± 204.2	0.706						
	65–75	673.9 ± 190.0	713.3 ± 229.1	0.753						
Carbohydrates (Kcal)	14–30	769.3 ± 297.2	715.2 ± 167.3	0.515						
	31–50	819.5 ± 230.1	679.4 ± 213.8	0.008						
	51–64	801.8 ± 283.0	609.5 ± 184.8	0.035						
	65–75	723.1 ± 227.1	665.3 ± 142.9	0.609						
Physical activity level (PAL)	14–30	1.68 ± 0.2	1.57 ± 0.03	0.039						
	31–50	1.63 ± 0.1	1.56 ± 0.02	0.006						
	51–64	1.65 ± 0.2	1.57 ± 0.02	0.066						
	65–75	1.64 ± 0.1	1.56 ± 0.01	0.101						

**Table 6:** Mean calorie intake (CI) according to the physical activity level (PAL) of patients with FSHD.

	Low PAL 1.57 (Men)–1.56 (Women)		*p* Value	Moderate PAL 1.78 (Men)–1.64 (Women)		*p* Value	High PAL 2.1 (Men)–1.82 (Women)	
	Men	Women		Men	Women		Men	Women
% population	76.2%	91.2%		16.7%	8%		7.1%	0%
CI	1858.47 ± 415.7	1615.6 ± 350.5	<0.001	1924.1 ± 510.6	1923.4 ± 446.2	0.998	2135.2 ± 549.6	

**Table 7:** Micronutrient and cholesterol intakes and their adequacy in 159 patients with FSHD.

	All Patients	Men		Women			Men			Women		
Parameters Recommendation	Mean ± SD	Mean ± SD	Median (Min–Max)	Mean ± SD	Median (Min–Max)	*p* Value	Adequate *n* (%)	Inadequate *n* (%)	Excessive *n* (%)	Adequate *n* (%)	Inadequate *n* (%)	Excessive *n* (%)
Vitamin C (mg) ≥110	83.4 ± 57.4	89.6 ± 62.4	75.9 (5–280.8)	89 ± 50.3	73.4 (18.0–271.3)	0.942	25 (29.8)	59 (70.2)		21 (28.4)	53 (71.6)	
Vitamin E (mg) M: ≥10.5 W: ≥9.9	8.5 ± 3.86	9.1 ± 4.1	8.5 (1.9–20.2)	7.8 ± 3.3	7.0 (2.6–18.8)	0.029	26 (31)	58 (69)		22 (29.7)	52 (70.3)	
Vit C/Vit E M: ≥7.3; W: ≥11.1	12.5 ± 10.2	11.5 ± 9.4	8.3 (1.3–45.0)	13.6 ± 11	10.2 (2.4–52.0)	0.189	47 (56)	37 (44)		38 (51.4)	36 (48.6)	
Cholesterol (mg) ≤300	295.2 ± 131.7	301.8 ± 130.3	279.7 (80.7–663.3)	287.7 ± 126.5	261.1 (16.0–703.3)	0.490	44 (52.4)		40 (47.6)	47 (63.5)		27 (36.5)
Vit E/Chol ≥0.03	0.036 ± 0.03	0.039 ± 0.04	0.027 (0.01–0.32)	0.035 ± 0.03	0.027 (0.008–0.16)	0.324	36 (42.8)	48 (57.2)		32 (43.2)	42 (56.8)	
Copper (mg) M: 1.25–5 W: 1–5	1.2 ± 0.46	1.3 ± 0.5	1.0 (0.5–3.0)	1.1 ± 0.4	1.0 (0.5–3.0)	0.011	33 (39.3)	51 (60.7)		68 (93.2)	5 (6.8)	
Zinc (mg) M: 14–25 W: 11–25	8.82 ± 3.5	9.5 ± 3.6	9.0 (3.0–24.0)	8 ± 3.0	8 (2.0–19.5)	0.005	11 (13.1)	73 (86.9)		13 (17.6)	61 (82.4)	
Cu/Zn M: 0.92–0.2 W: 0.90–0.2	0.15 ± 0.07	0.15 ± 0.07	0.14 (0.04–0.43)	0.15 ± 0.06	0.14 (0.07–0.33)	0.692	57 (67.8)	15 (17.8)	12 14.4	48 (64.9)	8 (10.8)	18 (24.3)
Selenium (µg) 70–300	124.6 ± 41.7	128.9 ± 41	127.0 (46.0–242.0)	119.6 ± 38.1	112.5 (54.0–229.5)	0.143	78 (92.9)	6 (7.1)		67 (90.6)	7 (9.4)	

**Table 8:** Micronutrient and cholesterol intakes and their adequacy in 159 patients with FSHD according to age.

		Men	Women			Men			Women	
Parameters/Recommendation	Ages (Years)	Mean ± SD	Mean ± SD	*p* Value	Adequate	Inadequate	Excess	Adequate	Inadequate	Excess
Vitamin C (mg) ≥110	14–30	73.9 ± 52.8	90.6 ± 62.7	0.397	4 (22.2)	14 (77.8)		7 (41.2)	10 (58.8)	
	31–50	75.2 ± 52.2	84.5 ± 46.1	0.415	6 (15.8)	33 (84.2)		8 (21.1)	30 (78.9)	
	51–64	122.2 ± 70.0	95.2 ± 53.4	0.238	13 (56.5)	10 (43.5)		3 (23.1)	10 (76.9)	
≥120	65–75	101.0 ± 80.8	98.8 ± 39.1	0.953	2 (33.3)	4 (66.7)		2 (33.3)	4 (66.7)	
Vitamin E (mg) M: ≥10.5; W: ≥9.9	14–30	7.8 ± 3.6	7.2 ± 4.3	0.619	3 (16.7)	15 (83.3)		3 (17.6)	14 (82.4)	
	31–50	9.6 ± 4.2	7.9 ± 2.6	0.040	15 (39.5)	23 (60.5)		9 (23.7)	29 (76.2)	
	51–64	8.8 ± 3.9	7.9 ± 3.1	0.508	2 (8.7)	21 (91.3)		13 (100)		
	65–75	10.7 ± 5.0	8.2 ± 5.2	0.405	1 (16.7)	5 (83.3)		1 (16.7)	5 (83.3)	
Vit C/Vit E M: ≥7.3; W: ≥11.1	14–30	11.3 ± 12.1	16.7 ± 15.1	0.255	7 (8.98)	11 (61.1)		6 (35.3)	11 (64.7)	
	31–50	9.0 ± 6.8	11.9 ± 8.3	0.099	11 (28.9)	27 (71.1)		18 (47.4)	20 (52.6)	
	51–64	15.9 ± 9.8	14.8 ± 13.6	0.785	17 (73.9)	6 (26.1)		8 (61.5)	5 (38.5)	
	65–75	10.7 ± 8.5	13.4 ± 4.8	0.513	3 (50)	3 (50)		6 (100)		
Cholesterol (mg) ≤300	14–30	275.1 ± 129.1	237.7 ± 147.9	0.430	10 (55.6)		8 (44.4)	13 (76.5)		4 (23.5)
	31–50	339.5 ± 135.1	292.6 ± 113.7	0.108	16 (42.1)		22 (57.9)	26 (68.4)		12 (31.6)
	51–64	271.2 ± 107.3	310.6 ± 103.9	0.291	14 (60.9)		9 (39.1)	6 (46.2)		7 (53.8)
	65–75	266.6 ± 155.4	348.9 ± 166.0	0.396	4 (66.7)		2 (33.3)	2 (33.3)		4 (66.7)
Vit E/Chol ≥0.03	14–30	0.04 ± 0.04	0.04 ± 0.04	0.640	4 (22.2)	14 (77.8)		8 (47.1)	9 (52.9)	
	31–50	0.04 ± 0.05	0.03 ± 0.02	0.441	13 (34.2)		25 (65.8)	15 (39.5)	23 (60.5)	
	51–64	0.05 ± 0.04	0.03 ± 0.032	0.260	18 (78.3)		5 (21.7)	2 (15.4)	11 (84.6)	
	65–75	0.02 ± 0.05	0.03 ± 0.01	0.085	3 (50)		3 (50)		6 (100)	
Copper (mg) M: 1.25–5; W: 1.1–5	14–30	1.14 ± 0.4	1.03 ± 0.3	0.367	4 (22.2)	14 (77.8)		2 (11.8)	15 (88.2)	
M: 1.9–5; W: 1.5–5	31–50	1.31 ± 0.4	1.07 ± 0.3	0.007	9 (23.7)	29 (76.3)		5 (13.2)	33 (86.8)	
M: 1.9–5; W: 1.5–5	51–64	1.46 ± 0.6	1.08 ± 0.3	0.034	8 (34.8)	15 (65.2)		3 (23.1)	10 (76.9)	
M: >1.6; W: >1.3	65–75	1.17 ± 0.3	1.83 ± 0.8	0.069	1 (16.7)	5 (83.3)		4 (66.7)	2 (33.3)	
Zinc (mg) M: 12; W: 10	14–30	8.6 ± 3.2	7.0 ± 2.2	0.108	3 (16.7)	15 (83.3)		1 (5.9)	16 (94.1)	
M: 12; W: 10	31–50	10.0 ± 3.3	8.3 ± 2.8	0.016	5 (13.2)	33 (86.8)		7 (18.4)	31 (81.6)	
M: 15–23; W: 15–23	51–64	9.7 ± 4.4	8.2 ± 4.2	0.327	1 (4.3)	22 (95.7)		1 (7.7)	12 (92.3)	
M: 15–23; W: 15–23	65–75	8.7 ± 1.6	8.9 ± 3.2	0.866		6 (100)			6 (100)	
Cu/Zn	14–30	0.15 ± 0.1	0.16 ± 0.1	0.551	8 (44.4)	4 (22.2)	6 (33.3)	9 (52.9)	8 (47.1)	
	31–50	0.14 ± 0.1	0.15 ± 0.1	0.753	15 (39.5)	23 (60.5)		10 (26.3)	28 (73.7)	
	51–64	0.17 ± 0.1	0.15 ± 0.0	0.367	9 (39.1)	14 (60.9)		5 (38.5)	8 (61.5)	
	65–75	0.09 ± 0.0	0.21 ± 0.1	0.066	2 (33.3)		4 (66.7)	4 (66.7)	2 (33.3)	
Selenium (µg) 70–300	14–30	115.9 ± 38.6	109.4 ± 33.6	0.602	16 (88.9)	2 (11.1)		14 (82.4)	3 (17.6)	
	31–50	135.5 ± 42.2	117.6 ± 34.3	0.046	37 (97.4)	1 (2.6)		36 (94.7)	2 (5.3)	
	51–64	122.7 ± 36.4	134.4 ± 46.7	0.408	20 (87)	3 (13)		12 (92.3)	1 (7.7)	
	65–75	151.6 ± 50.1	129.8 ± 50.1	0.468	6 (100)			5 (83.3)	1 (16.7)	

**Table 9:** Plasma concentrations of vitamins and minerals and their adequacy in 159 patients with FSHD.

	All Patients	Men		Women			Men			Women		
Parameters Normal Ranges	Mean ± SD	Mean ± SD	Median (Min–Max)	Mean ± SD	Median (Min–Max)	*p* Values	Within	Below	Above	Within	Below	Above
Vitamin C (µg/mL) ≥9.68–≤12.68	9.01 ± 3.7	8.3 ± 3.2	8.8 (0.9–14.85)	9.99 ± 3.8	10.4 (0.9–23.8)	0.003	23 (28)	53 (64.6)	6 (7.3)	27 (37.5)	29 (40.3)	16 (22.2)
Vitamin E (µg/mL) 8.6–19.24	13.1 ± 4.5	13.5 ± 5.	12.2 (6.9–37.2)	12.5 ± 3.2	12.4 (6.9–21.8)	0.171	67 (79.8)	8 (9.5)	9 (10.7)	65 (90.3)	4 (5.6)	3 (4.1)
Vit C/Vit E (µg/mL) 1–1.1	0.76 ± 0.37	0.68 ± 0.4	0.67 (0.08–1.6)	0.86 ± 0.4	0.86 (0.11–1.9)	0.003	7 (8.8)	64 (80)	9 (11.2)	6 (8.6)	46 (65.7)	18 (25.7)
Cholesterol (g/L) 1.60–2.21	1.94 ± 0.4	1.94 ± 0.4	1.91 (1.2–3.2)	1.95 ± 0.4	1.93 (0.9–3.0)	0.870	44 (53)	16 (19.3)	23 (27.7)	47 (63.5)	11 (11.9)	16 (21.6)
Vit E/Chol 4.4–7	6.7 ± 2.0	6.96 ± 2.3	6.5 (3.6–17.9)	6.4 ± 1.2	6.3 (4.5–10.5)	0.083	50 (61)	2 (2.4)	30 (36.6)	52 (73.2)		19 (26.8)
Copper (mg/L) M: 0.70–1.4 W: 0.80–1.55	1.03 ± 0.4	0.86 ± 0.2	0.85 (0.42–1.28)	1.21 ± 0.37	1.13 (0.57–2.4)	<0.001	69 (83.1)	14 (16.9)		57 (78.1)	4 (5.5)	12 (16.4)
Zinc (mg/L) M: 0.70–1.20 W: 0.70–1.30	0.93 ± 0.2	0.95 ± 0.17	0.96 (0.5–1.5)	0.90 ± 0.2	0.86 (0.55–1.5)	0.107	73 (86.9)	6 (7.1)	5 (6)	62 (84.9)	7 (9.6)	4 (5.5)
Cu/Zn 1–1.17	1.16 ± 0.5	0.94 ± 0.23	0.94 (0.39–1.4)	1.42 ± 0.57	1.26 (0.64–3.33)	0.001	23 (27.7)	48 (57.8)	12 (14.5)	15 (20.6)	13 (17.8)	45 (61.6)
Selenium (µg/L) 94–130	91.1 ± 31.4	93.3 ± 35.1	89.2 (39.8–301)	87.8 ± 22.1	88.1 (39.9–161)	0.203	25 (29.8)	52 (61.9)	7 (8.3)	24 (33.3)	45 (62.5)	3 (4.2)

**Table 10:** Plasma concentrations of vitamins and minerals and their adequacy in 159 patients with FSHD according to age.

		Men	Women		Men			Women		
Parameters Recommendations	Ages (Years)	Mean ± SD	Mean ± SD	*p* Value	Within *n*(%)	Below *n* (%)	Above *n* (%)	Within *n* (%)	Below *n* (%)	Above *n* (%)
Vitamin C	14–30	9.6 ± 2.2	10.7 ± 3.5	0.301	5 (29)	10 (59)	2 (12)	5 (31)	6 (38)	5 (31)
(µg/mL)	31–50	8.1 ± 3.6	9.3 ± 3.4	0.139	9 (25)	24 (67)	3 (8)	17 (46)	17 (46)	3 (8)
≥9.68–≤12.68	51–64	8.2 ± 2.9	9.4 ± 4.2	0.326	7 (30)	15 (65)	1 (4)	2 (15)	6 (46)	5 (38)
	65–75	6.4 ± 3.9	14.0 ± 4.9	0.014	2 (33)	4 (67)		3 (50)		3 (50)
Vitamin E (µg/mL)	14–30	10.4 ± 3.0	11.8 ± 3.3	0.185	13 (72)	5 (28)		15 (88)	1 (6)	1 (6)
8.6–19.24	31–50	14.1 ± 4.9	11.7 ± 2.5	0.009	29 (78)	2 (5)	6 (16)	34 (94)	2 (6)	
	51–64	15.0 ± 5.9	13.6 ± 3.4	0.448	19 (83)	1 (4)	3 (13)	12 (92)	1 (8)	
	65–75	12.9 ± 3.5	17.1 ± 2.7	0.042	6 (100)			4 (67)		2 (33)
Vit C/Vit E	14–30	0.9 ± 0.3	1.0 ± 0.4	0.946	3 (18)	9 (53)	5 (29)	2 (13)	8 (50)	6 (38)
(µg/mL) 1–1.1	31–50	0.6 ± 0.4	0.8 ± 0.3	0.015	2 (6)	30 (83)	4 (11)	2 (6)	25 (69)	9 (25)
	51–64	0.6 ± 0.3	0.8 ± 0.4	0.126	1 (5)	20 (91)	1 (5)	2 (17)	8 (67)	2 (17)
	65–75	0.5 ± 0.3	0.8 ± 0.3	0.117	1 (17)	5 (83)			5 (83)	1 (17)
Cholesterol (g/L)	14–30	1.6 ± 0.2	1.7 ± 0.2	0.191	9 (50)	9 (50)		12 (71)	5 (29)	
1.60–2.21	31–50	2.0 ± 0.4	1.9 ± 0.3	0.187	20 (56)	4 (11)	12 (33)	28 (74)	6 (16)	4 (11)
	51–64	2.1 ± 0.4	2.2 ± 0.2	0.595	11 (46)	3 (13)	10 (42)	6 (46)		7 (54)
	65–75	2.0 ± 0.3	2.6 ± 0.4	0.017	5 (83)		1 (17)	1 (17)		5 (83)
Vit E/Chol	14–30	6.7 ± 2.6	7.0 ± 1.4	0.732	14 (78)		4 (22)	10 (59)		7 (41)
4.4–7	31–50	7.2 ± 2.5	6.3 ± 1.1	0.059	22 (51)	1 (3)	13 (36)	28 (78)		8 (22)
	51–64	7.0 ± 1.8	6.0 ± 1.1	0.085	11 (50)	1 (5)	10 (45)	9 (75)		3 (25)
	65–75	6.2 ± 1.0	6.7 ± 1.1	0.525	3 (50)		3 (50)	5 (83)		1 (17)
Copper (mg/L)	14–30	0.9 ± 0.2	1.2 ± 0.4	0.003	16 (89)	2 (11)		11 (65)	1 (6)	5 (29)
M: 0.70–1.4	31–50	0.8 ± 0.2	1.2 ± 0.4	<0.001	30 (81)	7 (19)		29 (78)	3 (8)	5 (14)
W: 0.80–1.55	51–64	0.9 ± 0.2	1.2 ± 0.2	<0.001	18 (82)	4 (18)		11 (85)		2 (15)
	65–75	0.9 ± 0.2	1.1 ± 0.2	0.226	5 (82)	1 (17)		6 (100)		
Zinc (mg/L)	14–30	1.0 ± 0.1	0.9 ± 0.2	0.023	17 (94)		1 (6)	15 (88)	1 (6)	1 (6)
M: 0.70–1.20	31–50	0.9 ± 0.2	0.9 ± 0.2	0.133	30 (81)	4 (11)	3 (8)	32 (86)	4 (11)	1 (3)
W: 0.70–1.30	51–64	0.9 ± 0.2	1.0 ± 0.3	0.347	20(87)	2 (9)	1 (4)	10 (77)	1(8)	2 (15)
	65–75	0.9 ± 0.1	0.9 ± 0.2	0.912	6 (100)			6 (100)		
Cu/Zn	14–30	0.9 ± 0.2	1.4 ± 0.7	0.001	3 (17)	14 (78)	1 (6)	3 (18)	4 (24)	10 (59)
1–1.17	31–50	0.9 ± 0.2	1.5 ± 0.6	<0.001	14 (38)	21 (57)	2 (5)	7 (19)	5 (14)	25 (68)
	51–64	1.0 ± 0.3	1.3 ± 0.4	0.010	5 (23)	10 (45)	7 (32)	1 (8)	3 (23)	9 (69)
	65–75	1.0 ± 0.2	1.3 ± 0.6	0.326	1 (17)	3 (50)	2 (33)	4 (67)	1(17)	1 (17)
Selenium (µg/L)	14–30	75.7 ± 13.4	79.3 ± 17.8	0.503	1 (5)	18 (95)		2 (12)	15 (88)	
94–130	31–50	105.1 ± 44.3	92.7 ± 22.8	0.139	15 (41)	17 (46)	5 (14)	17 (47)	17 (47)	2 (6)
	51–64	93.4 ± 24.1	84.4 ± 26.0	0.303	8 (35)	13 (57)	2 (9)	3 (23)	9 (69)	1 (8)
	65–75	81.0 ± 28.9	89.4 ± 13.7	0.538	1 (17)	5 (83)		2 (33)	4 (67)	

**Table 11:** Plasma concentrations of vitamins and oligoelements and their adequacy in controls and in 32 patients with FSHD.

	Control Subjects	All Patients		Men (*n* = 20)		Women (*n* = 12)				Men			Women		
Parameters Normal Ranges	Mean ± SD	Mean ± SD	*p* Values	Mean ± SD	Median (Min–Max)	Mean ± SD	Median (Min–Max)	*p* Values	Cohen’d	Ade-quate	Inadequate	Exces-sive	Ade-quate	Inadequate	Excessive
Vitamin C (µg/mL) ≥9.68–≤ 2.68	11.6 ± 0.73	9.5 ± 3.3	<0.001	9.06 ± 2.8	9.5 (2.57–13.41)	10.3 ± 1.8	10.61 (6.4–12.94)	0.142	0.494	8 (40)	11 (55)	1 (5)	8 (66.7)	3 (25)	1 (8.3)
Vitamin E (µg/mL) 8.6–19.24	11.45 ± 0.64	12.2 ± 4.5	0.263	13.2 ± 4	12.1 (8.1–23.3)	10.6 ± 1.5	10.5 (7.1–13.2)	0.015	0.783	17 (85)	1 (5)	2 (10)	11 (91.7)	1 (8.3)	
Vit C/Vit E (µg/mL) 1–1.1	1.01 ± 0.06	0.84 ± 0.36	0.006	0.74 ± 0.3	0.78 (0.21–1.3)	1 ± 0.3	1.02 (0.49–1.43)	0.016	0.897	3 (15)	16 (80)	1 (5)	2 (16.7)	6 (50)	4 (33.3)
Cholesterol (g/L) 1.60–2.21	1.7 ± 0.11	1.95 ± 0.6	0.033	2 ± 0.4	2.0 (1.39–2.63)	1.87 ± 0.2	1.93 (1.44–2.12)	0.226	0.406	10 (50)	3 (15)	7 (15)	10 (83.3)	2 (16.7)	
Vit E/Chol 4.4–7	6.7 ± 0.13	6.3 ± 1.9	0.062	6.61 ± 2.3	6.22 (3.89–10.16)	5.7 ± 0.7	5.40 (4.86–6.92)	0.025	0.736	12 (60)	1 (5)	7 (35)	12 (100)		
Copper (mg/L) 0.94–1.20 M: 0.70–1.4 W: 0.80–1.55	1.02 ± 0.09	1.01 ± 0.4	0.892	0.87 ± 0.1	0.92 (0.55–1.08)	1.25 ± 0.27	1.23 (0.86–1.75)	<0.001	1.899	19 (95)	1 (5)		10 (83.3)		2 (16.7)
Zinc (mg/L) M: 0.70–1.20 W: 0.70–1.30	0.96 ± 0.11	0.83 ± 0.2	0.022	0.85 ± 0.16	0.87 (0.51–1.19)	0.80 ± 0.12	0.82 (0.55–1.03)	0.289	0.368	16 (80)	4 (20)		11 (91.7)	1 (8.3)	
Cu/Zn 1–1.17	1.07 ± 0.14	1.25 ± 0.5	0.049	1.0 ± 0.23	1.06 (0.76–1.36)	1.60 ± 0.43	1.49 (1.04–2.27)	0.001	1.958	11 (55)	6 (30)	3 (15)	2 (16.7)		10 (83.3)
Selenium (µg/L) 94–130	110.07 ± 8.0	107.3 ± 34.1	0.516	111.3 ± 27.9	104.25 (79–197.7)	100.7 ± 10.4	100.55 (76.0–113.8)	0.138	0.459	10 (50)	6 (30)	4 (20)	9 (75)	3 (25)	

**Table 12:** Plasma and urinary levels of antioxidants and oxidative stress markers and their adequacy in controls and in 32 patients with FSHD.

	Controls	All Patients		Men (*n* = 20)		Women (*n* = 12)				Men			Women		
Parameters Normal Ranges	Mean ± SD	Mean ± SD	*p* Values	Mean ± SD	Median (Min–Max)	Mean ± SD	Median (Min–Max)	*p* Values	Cohend	Within	Below	Above	Within	Below	Above
GSH tot (μmol/L) (717–1110)	828.1 ± 66.5	812.0 ± 192.4	0.644	808.7 ± 138.9	822.0 (572–1085)	817.5 ± 127.3	809.0 (565–994)	0.856	0.065	15 (75)	5 (25)		10 (83)	2 (17)	
GSH (μmol/L) (715–1090)	822.8 ± 67.3	799.2 ± 190.0	0.501	795.9 ± 142.7	818.6 (559.2–1083)	804.7 ± 116.0	799.41 (563.9–977.8)	0.852	0.065	14 (70)	6 (30)		10 (83)	2 (17)	
GSSG (μmol/L) (0.96–10) <4.85	2.6 ± 1.2	6.4 ± 10.1	0.050	6.4 ± 9	1.92 (0.53–28.3)	6.4 ± 12.4	2.53 (0.53–45)	0.995	0.003	16 (80	1 (5)	3 (15)	9 (75)	2 (17)	1 (8)
GSH/GSSG (111–747)	381 ± 186.7	468.9 ± 392.6	0.372	456.9 ± 396.5	422.5 (24–1357)	489.0 ± 395.9	348.5 (20–1268)	0.926	0.081	10 (50)	5 (25)	5 (25)	7 (58)	2 (17)	3 (25)
GSH–Px (UI/g Hb)	38.1 ± 5	48.5 ± 14.4	0.001	48.6 ± 13.3	46.3 (30.9–88.2)	48.2 ± 8.8	46.7 (33–68.5)	0.922	0.033						
CuZnSOD (UI/g Hb)	1178.6 ± 206.2	2199.6 ± 701.1	<0.001	2195.0 ± 483.1	2080.5 (1668–2933.8)	2206.1 ± 650.6	1955.6 (1523.8–3185.2)	0.966	0.020						
Lipid peroxides (μmol/L) (<432)	148 ± 31.3	523.4 ± 354.9	<0.001	329.7 ± 137.5	270 (160–608)	846.3 ± 357.3	822.5 (316–1543)	<0.001	2.131	14 (70)		6 (30)	2 (17)		10 (83)
Oxidized DNA (μg/L) (0–16)	8.08 ± 2.39	23.5 ± 12.6	<0.001	25.2 ± 13.7	21.3 (6.8–61.4)	20.9 ± 8.9	17.8 (11.8–38.8)	0.292	0.358	6 (32)		13 (68)	4 (33)		8 (67)
Oxidized DNA/creatinine (0–20)	ND	17.6 ± 7.8		17.7 ± 8.3	15.4 (7–33.6)	17.4 ± 5.3	15.6 (11.4–28.9)	0.907	0.039	12 (63)		7 (37)	9 (75)		3 (25)

**Table 13:** MRI evaluation of thigh parameters in patients with FSHD and healthy controls.

	Control Subjects (*n* = 7)			Patients with FSHD (*n* = 32)							
	All	Men (*n* = 4)	Women (*n* = 3)	All	Men (*n* = 20)	Women (*n* = 12)	Between Group Differences				
	Mean ± SD	Mean ± SD	Mean ± SD	Mean ± SD	Mean ± SD	Mean ± SD	All Controls vs. All FSHD	HC Men vs. HC Women	FSHD Men vs. FSHD Women	FSHD vs. HC Men	FSHD vs. HC Women
MVC_QD_	36.7 ± 4.9	37.3 ± 3	36.0 ± 7.1	16.5 ± 12.68	20.5 ± 14.15	9.9 ± 5.6	<0.001	0.088	0.006	<0.001	0.013
MVC _Q ND_	ND			13.8 ± 12.1	17.03 ± 14.06	8.63 ± 5.3			0.026		0.026
Dominant thigh											
MV_T_	2974.7 ** ± 880.7	3656.3 ± 615.6	2293.0 ± 407.6	1950.5 ** ± 1194.4	2256.3 ** ± 1275.9	1440.8 ± 869.2	0.037	0.040	0.040	0.026	0.040
FV_T_	173.1 ± 64.9	152.3 ± 41.8	193.9 ± 86.5	907.6 ± 639.4	991.7 ± 733.8	767.6 ± 433.2	<0.001	0.509	0.286	<0.001	<0.001
TV_T_	3147.7 * ± 843.4	3808.6 ± 579.1	2486.9 ± 364.4	2858.1 *** ± 891.3	3247.9 *** ± 807.5	2208.5 ± 613.1	0.467	0.037	<0.001	0.228	0.355
%FV_T_	6.1 ± 3.4	4.2 ± 1.7	8.0 ± 0.8	36.7 ** ± 27.9	34.9 ± 28.8	39.6 ± 27.3	<0.001	0.215	0.652	<0.001	0.002
%MV	93.9 ± 3.4	95.8 ± 1.7	92.0 ± 3.8	63.3 * ± 27.9	65.1 ** ± 28.8	60.4 ± 27.3	<0.001	0.215	0.652	<0.001	0.002
Non-dominant thigh											
MV_T_	2561.4 ± 621	3656.3 ± 615.6	2293.0 ± 407.6	2140.4 ± 1225.3	2540.4 ± 1234.6	1473.8 ± 907.1	0.074	0.040	0.009	0.056	0.041
FV_T_	190.8 ± 55.3	177.8 ± 59.7	199.5 ± 63.8	872.2 ± 611.9	948.2 ± 690.3	745.4 ± 451.8	<0.001	0.729	0.323	<0.001	0.002
TV_T_	2752.3 ± 593.2	3774.9 ± 504.9	2492.5 ± 364.7	3012.6 ± 952.0	3488.6 ± 794.1	2219.2 ± 605.3	0.727	0.027	<0.001	0.452	0.306
%FV_T_	7.4 ± 3.2	5.1 ± 2.0	8.3 ± 3.2	33.8 ± 26.9	30.6 ± 25.2	38.6 ± 28.3	<0.001	0.275	0.426	<0.001	0.004
%MV_T_	93.6 ± 3.2	94.9 ± 2.0	91.7 ± 3.2	66.2 ± 26.9	69.4 ± 25.2	60.9 ± 29.8	<0.001	0.275	0.426	<0.001	0.003

HC: healthy controls; ND: not determined; Significant difference between the dominant and non-dominant thigh: * *p* < 0.05, ** *p* < 0.01, and *** *p* < 0.001.

**Table 14:** Correlations in patients with FSHD.

Parameter 1	Parameter 2	All Patients (*n* = 159)		Men (*n* = 85)		Women (*n* = 74)		All Patients (*n* = 32)		Men (*n* = 20)		Women (*n* = 12)	
		r	*p*	r	*p*	r	*p*	r	*p*	r	*p*	r	*p*
Correlations between calorie intake and dietary energy parameters and micronutrient intakes													
CI	Proteins (g/kg body weight)	0.316	<0.001	0.531	<0.001	0.415	<0.001						
CI	Protein/energy %	−0.316	<0.001	−0.372	<0.001	−0.296	<0.001	−0.513	0.003	−0.456	0.043		
CI	Proteins (kcal)	0.489	<0.001	0.494	<0.001	0.557	<0.001	0.544	0.002	0.576	0.008		
CI	Lipids (kcal)	0.637	<0.001	0.731	<0.001	0.816	<0.001	0.788	<0.001	0.803	<0.001	0.891	<0.001
CI	Carbohydrates (kcal)	0.776	<0.001	0.744	<0.001	0.776	<0.001	0.85	<0.001	0.786	<0.001	0.691	0.016
CI	Vitamin E	0.470	<0.001	0.463	<0.001	0.421	<0.001	0.566	<0.001	0.559	0.010		
CI	VitC/VitE ratio	−0.245	0.003	−0.238	0.036								
CI	Cholesterol	0.317	<0.001	0.357	0.001	0.233	0.047	0.479	0.007	0.472	0.035	0.709	0.013
CI	Selenium	0.353	<0.001	0.365	0.001								
CI	Copper	0.429	<0.001	0.382	<0.001	0.385	<0.001	0.539	0.002	0.591	0.006		
CI	Zinc	0.492	<0.001	0.321	0.004	0.581	<0.001	0.407	0.023	0.426	0.05		
CI	Cu/Zn ratio					−0.281	0.016						
Correlations between micronutrient intakes													
Copper	Zinc	0.35	<0.001	0.268	0.014	0.379	<0.001						
Cu/Zn ratio	Copper	0.43	<0.001	0.519	<0.001	0.371	0.001						
Cu/Zn ratio	Zinc	−0.648	<0.001	−0.641	<0.001	−0.671	<0.001						
Copper	Selenium	0.288	<0.001	0.252	0.021	0.27	0.020						
Zinc	Selenium	0.296	<0.001	0.271	0.013	0.267	0.022						
Correlations between age and micronutrients intakes													
Age	Vitamin C	0.164	0.040	0.282	0.010								
Age	VitC/VitE ratio			0.265	0.015								
Correlations between age and plasma concentration of micronutrients													
Age	Vitamin C			−0.233	0.035								
Age	Vitamin E	0.358	<0.001	0.324	0.003	0.357	0.002						
Age	VitC/VitE ratio	−0.266	0.001	−0.338	0.002								
Age	Cholesterol	0.469	<0.001	0.380	<0.001	0.551	<0.001						
Correlations between PAL and dietary energy and micronutrient intakes													
PAL	CI	0.302	0.001					0.415	0.020			0.671	0.021
PAL	Protein/energy %											−0.671	0.021
PAL	Lipid /energy %									−0.495	0.026		
PAL	Protide (kcal)	0.215	0.019					0.434	0.015				
PAL	Carbohydrates (kcal)	0.271	0.003					0.388	0.031			0.671	0.021
PAL	Copper intake	0.195	0.033	0.306	0.016								
PAL	Zinc intake	0.270	0.003	0.282	0.027								
PAL	Selenium intake	0.204	0.026										
Correlations between PAL and plasma concentration of micronutrients													
PAL	Plasma Copper	−0.355	<0.001			0.281	0.034						
PAL	Plasma Cu/Zn	−0.351	<0.001					−0.387	0.032				

**Table 15:** Correlations in 32 patients with FSHD.

		ALL (*n* = 32)		Men (*n* = 20)		Women (*n* = 12)	
		r	*p*	r	*p*	r	*p*
Correlations between calorie intake and muscle parameters							
CI	MV DOM	0.396	0.027			0.718	0.011
CI	MV ND	0.349	0.050			0.655	0.026
CI	%MV DOM	0.384	0.033			0.800	0.001
CI	%MV ND	0.347	0.050			0.745	0.007
CI	FV DOM					−0.800	0.001
CI	FV ND					−0.764	0.005
CI	%FV DOM	−0.384	0.032			−0.800	0.001
CI	%FV ND	−0.347	0.050			−0.745	0.007
CI	TV DOM	0.369	0.041				
CI	TV ND	0.374	0.038				
CI	PAL	0.415	0.020			0.671	0.021
Correlations between muscle parameters and blood concentrations of micronutrients and oxidative stress markers							
Vitamin E	FV DOM					−0.664	0.017
Vitamin E	%FV DOM					−0.629	0.026
Vitamin E	%MV DOM					0.629	0.026
Vit C/E ratio	TV DOM	−0.439	0.012				
Vit C/E ratio	TV ND	−0.467	0.007				
VitE/chol	MV ND					0.657	0.019
VitE/chol	TV DOM					0.629	0.026
VitE/chol	TV ND	0.414	0.019			0.776	0.002
Copper	MV DOM					0.615	0.031
Cu/Zn ratio	%MV DOM			0.450	0.046		
GSH total	%MV DOM			0.445	0.048		
GSH	%MV DOM			0.447	0.048		
GSH total	%FV DOM			−0.445	0.048		
GSH	%FV DOM			−0.447	0.048		
CuZnSOD	MV DOM					0.661	0.033
CuZnSOD	MV ND					0.697	0.022
CuZnSOD	FV DOM					−0.697	0.022
CuZnSOD	FV ND					−0.673	0.029
CuZnSOD	TV DOM					0.648	0.038
CuZnSOD	%MV DOM					0.733	0.013
CuZnSOD	%MV ND					0.673	0.029
CuZnSOD	%FV DOM					−0.733	0.013
CuZnSOD	%FV ND					−0.673	0.029
Lipid peroxides	MV DOM	−0.427	0.017	−0.498	0.025		
Lipid peroxides	MV ND	−0.471	0.007	−0.540	0.014		
Lipid peroxides	TV DOM	−0.584	<0.001	−0.567	0.009		
Lipid peroxides	TV ND	−0.615	<0.001	−0.519	0.019		
Lipid peroxides	FV ND			0.463	0.039		
Lipid peroxides	%MV DOM			−0.450	0.046		
Lipid peroxides	%MV ND			−0.535	0.015		
Lipid peroxides	%FV DOM			0.450	0.046		
Lipid peroxides	%FV ND			0.535	0.015		
oxDNA/creat ratio	MV DOM	−0.635	<0.001	−0.725	<0.001		
oxDNA/creat ratio	MV ND	−0.566	<0.001	−0.674	<0.001		
oxDNA/creat ratio	%MV DOM	−0.637	<0.001	−0.735	<0.001		
oxDNA/creat ratio	%MV ND	−0.584	<0.001	−0.642	0.003		
oxDNA/creat ratio	FV DOM	0.633	<0.001	0.682	<0.001		
oxDNA/creat ratio	FV ND	0.585	<0.001	0.654	0.002		
oxDNA/creat ratio	%FV DOM	0.637	<0.001	0.735	<0.001		
oxDNA/creat ratio	%FV ND	0.584	<0.001	0.642	<0.001		
oxDNA/creat ratio	TV DOM	−0.362	0.045	−0.532	0.019		
oxDNA/creat ratio	TV ND			−0.474	0.039		
Correlations between PAL and muscle parameters							
PAL	TV ND	0.365	0.040				
PAL	FV DOM			−0.481	0.031		
PAL	FV ND			−0.458	0.042		
PAL	%FV DOM			−0.435	0.05		
PAL	%FV ND			−0.461	0.040		
PAL	%MV DOM			0.435	0.05		
PAL	%MV ND			0.461	0.040		
Correlations between blood concentration of micronutrients and oxidative stress markers							
Copper	Cu/Zn ratio	0.641	<0.001			0.741	0.005
Vitamin C	Copper	0.411	0.019				
Vitamin C	Cu/Zn ratio	0.445	0.012				
Lipid peroxides	Copper	0.729	<0.001			0.936	<0.001
Lipid peroxides	Cu/Zn ratio	0.502	0.005			0.855	<0.001
Vitamin E	Cholesterol	0.588	<0.001	0.637	0.002		
Vitamin E	VitC/E ratio	−0.742	<0.001	−0.674	0.001	−0.692	0.011
Vitamin C	VitC/E ratio	0.791	<0.001	0.732	<0.001	0.895	<0.001
VitC/E ratio	Cholesterol	−0.422	0.016				
VitC/E ratio	Copper	0.376	0.034				
VitC/E ratio	Lipid peroxides	0.433	0.015				
GSH tot	GSH	0.991	<0.001	0.988	<0.001	0.993	<0.001
GSH tot	GSSG					0.741	0.005
GSH tot	GSH/GSSG ratio					−0.657	0.019
GSH	GSH/GSSG ratio			0.454	0.044	−0.622	0.028
GSSG	GSH					0.720	0.007
GSSG	GSH/GSSG ratio	−0.985	<0.001	−0.976	<0.001	−0.972	<0.001
Copper	GSH/GSSG ratio			0.655	0.002		
Copper	GSSG			−0.653	0.002		
oxDNA/creat ratio	GSH tot			−0.486	0.034		
oxDNA/creat ratio	Cu/Zn ratio			0.551	0.014		
oxDNA/creat ratio	GSH	−0.373	0.039	−0.479	0.037		
GSH	Lipid peroxides					0.618	0.039

## Data Availability

Since the participants only gave consent to report the summary of data, no individual data can be shared publicly.

## Supplementary Materials

The following supporting information can be downloaded at: https://www.mdpi.com/article/10.3390/nu15071673/s1, Table S1: Demographic. clinical and anthropometric characteristics of patients with FSHD (*n* = 32) and healthy controls (*n* = 7); Table S2: Energy. macronutrient energy intakes and micronutrient intakes in 32 patients with FSHD.
