# Supplementary material for: Nutritional Status of Patients with Facioscapulohumeral Muscular Dystrophy
Source: Nutrients. 2023 Mar 30;15(7):1673. doi: 10.3390/nu15071673 (PMC10096775; doi:10.3390/nu15071673)
Supplement: Supplementary file 1 [file nutrients-15-01673-s001.zip › nutrients-2259669-supplementary.pdf]

Supplemental Table S1. Demographic. clinical and anthropometric characteristics of patients with FSHD (n=32) and healthy controls (n=7). SD. standard deviation; BMI. body mass index.

	**Control Subjects**	**All Patients (n=32)**	**Men (n=20)**	**Women (n=12)**		
	**Mean SD**	**Mean SD**	**Mean SD**	**Mean SD**	**p Values**	**d Cohen**
Age (years)	42.1 ±9.8	39.6 ±11.6	40.8 ±11.0	37.8 ±6.7	0.346	0.311
D4Z4 (repeat units)		6.1 ±2.1	6.3 ±1.6	5.6 ±2.1	0.381	0.402
Body weight (kg)	70.1 ±11.5	69.7 ±24.7	77.8 ±10.8	56.2 ±21.9	0.006	1.368
Body height (cm)	172.7 ±10.6	172.7 ±42.2	177.2 ±6.9	164.5 ±7.5	<0.001	1.775
BMI (kg/m^2)	23.4 ±2.6	24.0 ±6.9	24.8 ±3.3	22.5 ±4.6	0.161	0.602

Supplemental Table S2. Energy. macronutrient energy intakes and micronutrients intakes in 32 patients with FSHD.

	**All patients (n=32)**	**Men (n=20)**	**Women (n=12)**		
	Mean SD	Mean SD	Mean SD	p values	d cohen
Calorie intake (CI)(kcal)	1823.6 ±589.5	1961.7 ±381.2	1572.5 ±321.9	0.006	1.079
Protein/energy ratio (%) 10 – 20	19.1 ±5.9	18.7 ±3.3	19.7 ±4.9	0.537	0.264
Protein intake (g/kg body weight/day): 1.2 -2	1.2 ±0.4	1.2 ±0.3	1.2 ±0.3	0.481	0.281
Fat/energy ratio (%) 35 – 40	37.9 ±10.6	37.1 ±5.2	39.2 ±6.0	0.341	0.382
Carbohydrate/energy ratio (%) 40 – 55	43.1 ±12.4	44.2 ±6.1	41.0 ±8.3	0.290	0.447
Protides (Kcal)	330.8 ±105.0	351.9 ±68.3	292.3 ±57.9	0.017	0.921
Lipides (Kcal)	672.7 ±243.0	713.6 ±193.9	598.4 ±151.8	0.079	0.642
Glucides (Kcal)	770.8 ±298.4	844.2 ±213.7	637.2 ±236.8	0.026	0.930
Physical activity level (PAL)	1.60 ±0.40	1.62 ±0.13	1.57 ±0.03	0.162	0.418
Micronutrient and cholesterol intakes					
Vitamin C (mg) ≥ 110	70.4 ±45.4	65.5 ±44.2	79.3 ±42.8	0.405	0.316
Vitamin E (mg) M: ≥10.5 ; W: ≥9.9	8.6 ±3.5	9.2 ±3.1	7.6 ±2.4	0.123	0.554
Vit C/Vit E M: ≥7.3 ; W: ≥11.1	8.9 ±5.8	7.9 ±5.8	10.9 ±4.7	0.132	0.553
Cholesterol (mg) ≤300	340.8 ±147.2	345.1 ±117.4	332.9 ±145.6	0.815	0.095
Vit E/Chol ≥0.03	0.028 ±0.013	0.029 ±0.012	0.025 ±0.010	0.348	0.340
Copper (mg) M: 1.25 – 5 ; W: 1 - 5	1.2 ±0.5	1.3 ±0.4	1.1 ±0.3	0.248	0.400
Zinc (mg) M: 14 – 25 ; W: 11 - 25	9.4 ±3.8	9.9 ±3.2	8.5 ±2.9	0.232	0.452
Cu/Zn M: 0.92-0.2; W: 0.90-0.2	0.140 ±0.066	0.140 ±0.062	0.142 ±0.053	0.899	0.046
Selenium (μg) 70 - 300	123.8 ±48.5	131.6 ±42.5	109.7 ±29.6	0.105	0.572
